# Intestinal dual-specificity phosphatase 6 regulates the cold-induced gut microbiota remodeling to promote white adipose browning

**DOI:** 10.1038/s41522-024-00495-8

**Published:** 2024-03-13

**Authors:** Pei-Chen Chen, Tzu-Pei Tsai, Yi-Chu Liao, Yu-Chieh Liao, Hung-Wei Cheng, Yi-Hsiu Weng, Chiao-Mei Lin, Cheng-Yuan Kao, Chih-Cheng Tai, Jhen-Wei Ruan

**Affiliations:** 1 Department of Medical Laboratory Science and Biotechnology, College of Medicine, National Cheng Kung University, Tainan, 70101 Taiwan; 2 Institute of Basic Medical Sciences, College of Medicine, National Cheng Kung University, Tainan, 70101, Taiwan; 3 Institute of Population Health Sciences, National Health Research Institutes, Miaoli, 35053 Taiwan; 4 Immunology Research Center, National Health Research Institutes, Zhunan, Miaoli, 35053 Taiwan; 5 Tainnovation Inc., Los Gatos, CA 95032 USA; 6 Research Center for Medical Laboratory Biotechnology, College of Medicine, National Cheng Kung University, Tainan, 70101 Taiwan

**Keywords:** Microbiome, Microbial ecology

## Abstract

Gut microbiota rearrangement induced by cold temperature is crucial for browning in murine white adipose tissue. This study provides evidence that DUSP6, a host factor, plays a critical role in regulating cold-induced gut microbiota rearrangement. When exposed to cold, the downregulation of intestinal DUSP6 increased the capacity of gut microbiota to produce ursodeoxycholic acid (UDCA). The DUSP6-UDCA axis is essential for driving *Lachnospiraceae* expansion in the cold microbiota. In mice experiencing cold-room temperature (CR) transitions, prolonged DUSP6 inhibition via the DUSP6 inhibitor (E/Z)-BCI maintained increased cecal UDCA levels and cold-like microbiota networks. By analyzing DUSP6-regulated microbiota dynamics in cold-exposed mice, we identified *Marvinbryantia* as a genus whose abundance increased in response to cold exposure. When inoculated with human-origin *Marvinbryantia formatexigens*, germ-free recipient mice exhibited significantly enhanced browning phenotypes in white adipose tissue. Moreover, *M. formatexigens* secreted the methylated amino acid Nε-methyl-L-lysine, an enriched cecal metabolite in *Dusp6* knockout mice that reduces adiposity and ameliorates nonalcoholic steatohepatitis in mice. Our work revealed that host-microbiota coadaptation to cold environments is essential for regulating the browning-promoting gut microbiome.

## Introduction

Metagenomic plasticity is critical for the adaptation capabilities of host to environmental variations^1–5^. When coevolved with microbiota, animals may gather environmental information by monitoring the changes in microbial cues for regulating metabolism^6,7^. However, it is uncertain whether the intensity and speed of changes in gut microbiota can efficiently counteract rapid environmental shifts^1^. Studies have suggested that diet and environmental factors dominate over genetic factors in affecting the composition of the microbiota^8,9^. However, host genetics are also considered to play critical roles in shaping the taxonomic composition of the gut microbiota^10,11^. The regulation and modification of the gastrointestinal mucus barrier have also been suggested to be responsive to the variations in diet, the environment, and stresses that contribute to the mucosal-to-luminal microbiota changes^12^. Therefore, it has been suggested that the beneficial effects of a microbiome should be investigated by considering the interaction between host control and microbial competition^13^.

During evolution, most mammalian species may repeatedly face the risk of the cold challenge. To adapt to this survival threat, mammals can maintain energy homeostasis and core body temperature by increasing the activity of brown adipose tissue (BAT) and beige adipocytes in white adipose tissue (WAT), a process that is often referred to as “beiging” or “browning”^14,15^. WAT is a dynamic organ whose function constantly changes its function between energy storage and lipid mobilization according to the dynamic variations in nutrient availability and the environment^15,16^. Unlike BAT, which has a constant thermogenic function, the dynamic browning of WAT is controlled by the differentiation of beige adipocytes accompanied by the increased expression of the mitochondrial uncoupling protein 1 (UCP1)^17,18^. Recently, a study has shown that cold stress can activate WAT browning in a gut-microbiome-dependent manner^19^. The conversion of cholesterol to bile acids triggered by cold exposure plays a crucial role in shaping the gut microbiome and facilitating adaptive thermogenesis^20^. Cold-induced alterations in mucosal and cecal microbiota could further contribute to increases in circulating amino acids and bile acids^21^. This bidirectional interaction between bile acids and gut microbiota significantly influences the host’s metabolic adaptation to cold challenges. In cold-exposed mice, a decreased abundance of *Akkermansia muciniphila* is a critical feature of microbiota remodeling^19^. Supplementation of cold-treated mice with *A. muciniphila* reversed the cold-induced intestinal absorptive phenotype but not WAT browning, suggesting that the browning-promoting function of the cold microbiota may be contributed by other species^19^. Currently, the specific browning-associated species or populations in cold microbiota remain unidentified.

Dual-specificity phosphatase 6 (DUSP6), a member of the MAPK phosphatase family, negatively regulates the target kinases by dephosphorylation of serine/threonine and tyrosine residues^22^. As a MAPK signaling regulator, DUSP6 has been shown to both promote and inhibit the development and progression of various diseases, including osteoporosis, retinal degeneration, neurological disorders and cancers^23–26^. In mice, *Dusp6* expression can increase fasting blood glucose levels by enhancing hepatic gluconeogenesis^27^. In a microbiota-dependent manner, *Dusp6* deficiency has been shown to protect mice from diet-induced obesity by harboring a unique leanness-associated gut microbiota^28^. In evolution, hosts consistently carrying a leanness-associated microbiota should be outcompeted by hosts harboring a microbiota tending to produce maximal metabolic output^29^. Therefore, we hypothesized that the *Dusp6*-mediated regulation of the leanness-associated gut microbiota might be a transient phenomenon for helping hosts adapt to environmental variations. Here we showed that in conventionally-raised (CONV-R) wild-type (WT) mice, the downregulation of intestinal DUSP6 is critical for modulating the browning function of a cold-adapted microbiota. During cold exposure, the reduced expression of intestinal DUSP6 coordinates the O-linked glycosylation of mucins and the production of microbial UDCA to trigger the browning-associated microbiota rearrangements. This study identified the DUSP6*-*UDCA axis as a molecular switch for microbiota-mediated adipose browning for rapid phenotypic adaptation to cold exposure.

## Results

### Intestinal *Dusp6* contributes to the regulation of cold-induced WAT browning

**Fig. 1:**
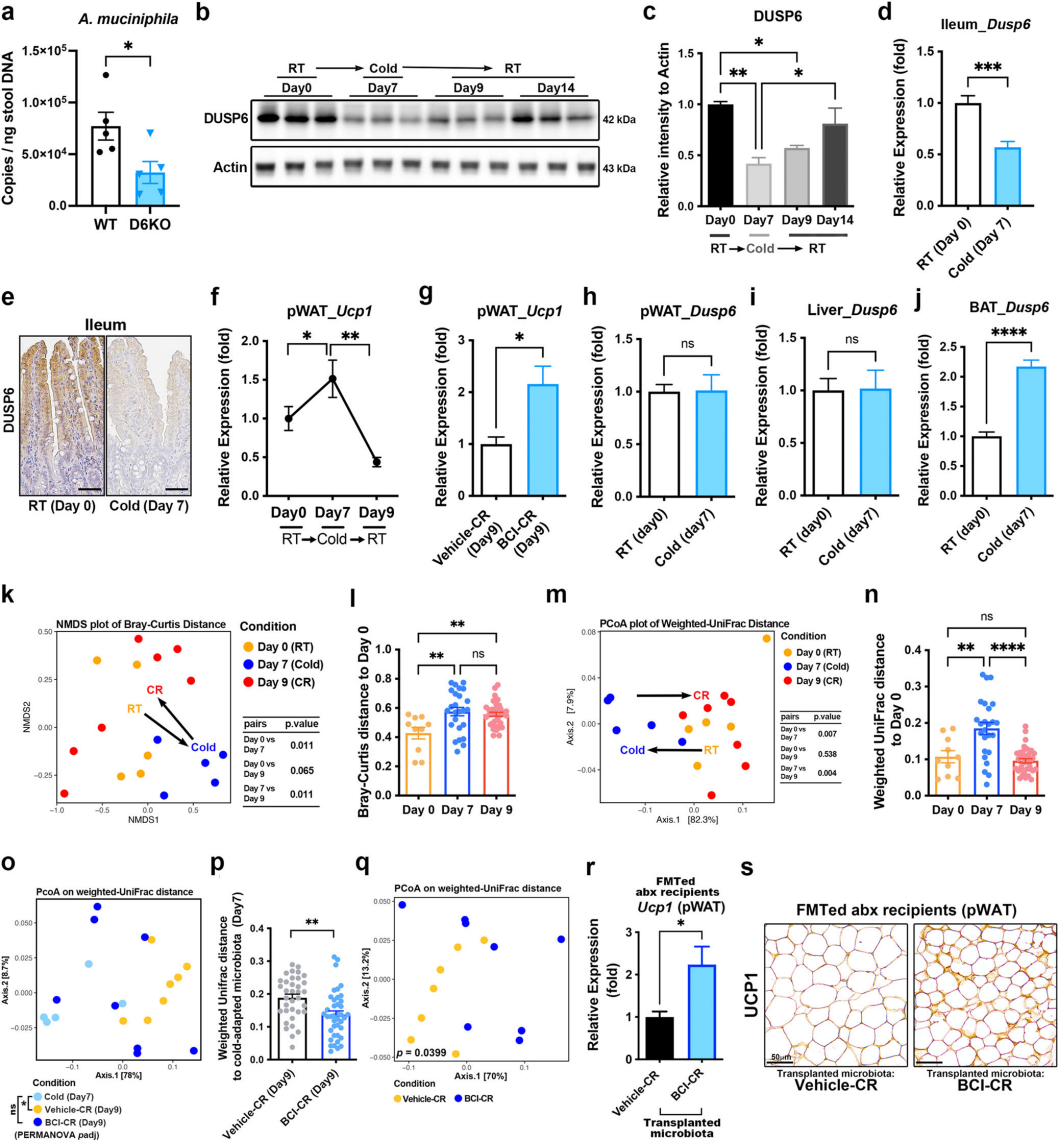
Downregulation of intestinal DUSP6 is a cold-induced phenomenon in mice. **a** qPCR analyses on 16S rRNA gene copies of *A. muciniphila* in fecal DNA of WT and *Dusp6*-deficient mice. *N* = 5 mice of each group. **b** Immunoblot analysis of DUSP6 protein in the ileum of mice before, during, and after cold treatment. Each column represents a protein lysate of ileum collected from an individual mouse (**b**). **c** The pixel intensities of each DUSP6 band in (**b**) were quantified and normalized to Actin protein. *N* = 3 mice of each group. **d** qRT-PCR analysis of *Dusp6* mRNA expression in ileum isolated from mice with or without cold exposure. *N* = 8 mice for each group. **e** Immunohistochemistry analysis of DUSP6 in the ileum of mice with (7 days) or without (RT) cold exposure. Scale bar, 50 μm. **f** qRT-PCR analysis of *Ucp1* mRNA expression in pWAT of mice during RT-cold-RT transition. *N* = 5–8 mice for each group. **g** qRT-PCR analysis of *Ucp1* mRNA expression in pWAT of vehicle- or BCI-treated mice underwent CR transition. *N* = 8 mice for each group. **h**–**j** qRT-PCR analysis of *Dusp6* mRNA expression in pWAT (**h**), liver (**i**), and BAT (**j**) isolated from mice with or without cold exposure. *N* = 8 mice for each group. **k**, **m** NMDS plot based on Bray-Curtis distance (**k**) or PCoA plot based on weighted UniFrac distance (**m**) of cold-adapted gut microbiota. Black arrows indicate the changing trend of microbiota during the RT (Day 0)-cold (Day 7: mice under cold exposure for 7 days)-RT (Day 9 (CR): 2 days after the end of cold exposure) transition. *N* = 5–7 mice for each group. *P-*value is calculated by pairwise PERMANOVA. **l**, **n** Box plots of between-group distances for Bray-Curtis distance (**l**) in (**k**) or weighted UniFrac distance (**n**) in (**m**). **o** PCoA plot of gut microbiota based on weighted UniFrac distance of mice underwent CR transition. Day 7: *N* = 5 mice; Day 9: Vehicle-CR and BCI-CR, *N* = 7-8 mice of each group. **p** Box plots of between-group weighted-UniFrac distances between microbial communities of Vehicle-CR or BCI-CR and cold-exposed (Day 7) mice presented in (**o**). **q** PCoA plot of Vehicle-CR and BCI-CR microbiota based on weighted UniFrac distance. *P-*value is calculated by PERMANOVA. *N* = 7-8 mice of each group. **r**, **s** qRT-PCR analysis **r** and immunohistochemistry analysis (**s**) of *Ucp1* expressions in pWAT of abx recipient mice transplanted with Vehicle-CR or BCI-CR microbiota. *N* = 8 mice of each group. **a**, **d**, **g**, **h**, **i**, **j**, **p**, **r** Data are presented as the mean ± SEM. ns, non-significant; *P < 0.05; **P < 0.01; ***P < 0.001; ****P < 0.0001 according to unpaired *t*-test. **c**, **f**, **l**, **n** Data are presented as the mean ± SEM. ns, non-significant; *P < 0.05; **P < 0.01; ****P < 0.0001 according to One-Way ANOVA analysis and Tukey post-hoc test.

In our previous study, we demonstrated that the gut microbiota of *Dusp6*-deficient (D6KO) mice has a dramatic effect on protecting fecal microbiota transplanted (FMTed) germ-free mice from diet-induced obesity^28^. However, the specific bacterial species or consortia that can exert obesity-resistant effects in *Dusp6*-deficient mice have not been identified. Interestingly, by performing qPCR analyses on fecal DNA samples, we noticed that *Dusp6*-deficient mice had a lower abundance of *A. muciniphila* in the gut microbiota when compared to WT mice (Fig. 1a). This observation led us to investigate whether *Dusp6* plays a role in shaping gut microbiota into a cold-adapted composition, which has also been shown to have a significant feature of reduced *A. muciniphila*^19^. To further determine whether the downregulation of intestinal DUSP6 is a cold-induced phenomenon in mice, we assessed changes in ileal DUSP6 expression in mice before, during, and after cold exposure. The results showed that cold stress significantly downregulated ileal DUSP6 expression after 7 days of cold stimulation (Fig. 1b, c). The 7 day cold exposure did not result in a significant change in the body weight of the exposed mice (Supplementary Fig. 1a). The downregulation of ileal DUSP6 was reversed after the cold stress was removed (Fig. 1c). The cold-induced decrease in *Dusp6* expression was further verified through qRT-PCR and immunohistochemistry analyses (Fig. 1d, e). During the cold (6 °C)-room temperature (RT) transition (CR transition), cold-induced *Ucp1* expression in perigonadal WAT (pWAT) was rapidly reversed after the mice were returned to RT condition (Fig. 1f). When mice undergoing CR transition were treated with the DUSP6 inhibitor (E/Z)-BCI (BCI) (IP injection of BCI before the end of cold treatment) to maintain the suppressed state of DUSP6 after the cold stress was removed (daily IP injection of BCI during the CR period) (Supplementary Fig. 1b), BCI treatment significantly maintained the *Ucp1* expression in the pWAT of BCI-CR mice even when cold stimulation was terminated (Fig. 1g). No significant difference in body weight was observed between the Vehicle-CR and BCI-CR mice (Supplementary Fig. 1c). To confirm the off-target effects of BCI on adipose browning, we administered BCI treatment to D6KO mice using the same protocol as for BCI-CR mice. The results showed that BCI treatment did not significantly affect *Ucp1* expression in the pWAT, inguinal WAT (iWAT) or BAT of D6KO mice (Supplementary Fig. 1d–f). Through qRT-PCR analyses, we confirmed that *Dusp6* expression was not reduced in the pWAT, liver, or BAT of mice exposed to cold conditions (Fig. 1h–j). Conversely, we observed a significant cold-induced increase in *Dusp6* expression in BAT (Fig. 1j). These findings indicate that the cold-induced decrease in *Dusp6* expression is specific to intestine.

By monitoring the changes in the gut microbiota during the RT-cold-RT transition (Supplementary Fig. 1b), reversible cold-induced changes in the gut microbiota were observed in cold-exposed mice by Bray-Curtis distance and weighted UniFrac distance (Fig. 1k–n). The similarity analysis demonstrated that the BCI-CR (Day 9; 2 days after the termination of cold treatment with daily BCI treatment) microbiota was more similar to the cold-adapted (Day 7) microbiota than the vehicle-CR (Day 9) microbiota on weighted UniFrac distance (Fig. 1o, p). Significant differences were observed between the BCI-CR and vehicle-CR microbiota according to the weighted UniFrac distance (Fig. 1q). By ordination analysis, we found that the DUSP6-inhibition during the CR transition primarily affected the shifts in the *Verrucomicrobiae* and *Clostridia* classes in the CR microbiota (Supplementary Fig. 1g). Furthermore, to confirm that DUSP6-inhibition indeed preserves the browning-associated feature of the cold microbiota to maintain the WAT browning phenotype when mice are returned to the RT condition, we subsequently transplanted the BCI-CR microbiota into mice with antibiotic-depleted gut microbiota (abx: gut microbiota was depleted by a 21 day course of antibiotic cocktail) (Supplementary Fig. 1h). One day after a single FMT, there was no significant difference in body weight between the recipients receiving the Vehicle-CR and BCI-CR microbiota (Supplementary Fig. 1i). Notably, the BCI-CR microbiota demonstrated an enhanced ability to induce *Ucp1* expression in WAT (Fig. 1r, s). The findings suggest that the BCI-CR microbiota maintained its ability to promote browning, and the WAT quickly responded to the browning signal after single FMT. In contrast, the vehicle-CR microbiota lost their ability to promote browning. Additionally, the inhibition of DUSP6 may be linked to changes in the *Clostridia*-*Verrucomicrobiae* composition within the cold-adapted gut microbiota.

### DUSP6 modulates the compositional shift in the cold gut microbiota

**Fig. 2:**
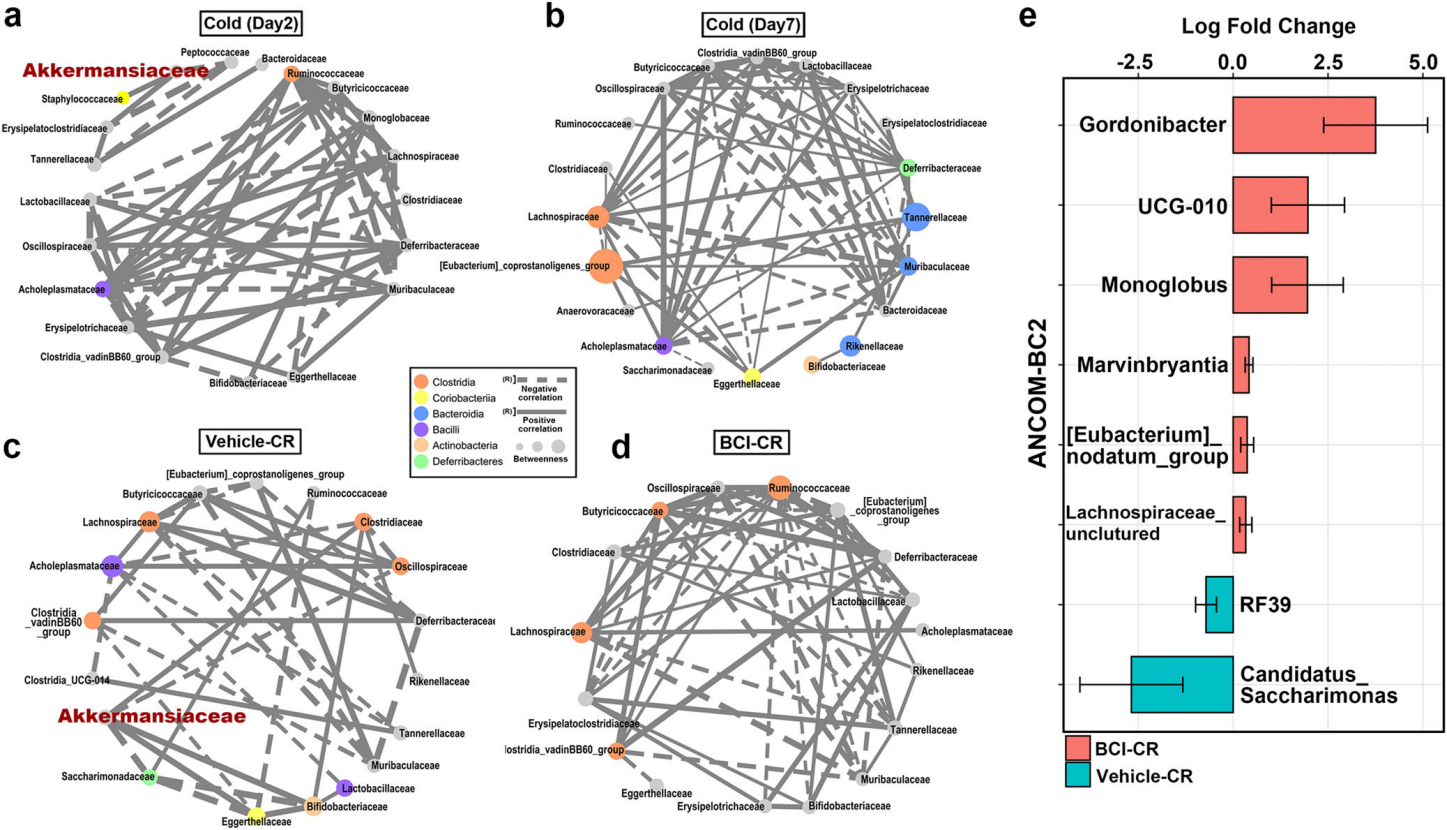
DUSP6 influences the rearrangements of cold microbiota. **a**–**d** Co-occurrence networks of cold (Day 2) (**a**), cold (Day 7) (**b**), Vehicle-CR (**c**) and BCI-CR (**d**) microbiota were constructed based on Spearman’s rank correlation. The networks displayed edges exhibiting a Spearman’s rank correlation exceeding 0.6 with statistical significance at a 0.05 *P*-value threshold. The thickness of the edges is determined by the correlation index. Solid lines represent positive correlations, and dashed lines represent negative correlations. Nodes are sized by the betweenness. The node with a betweenness >10 was colored by class. **e** The differentially abundant genera (*P* < 0.05) were identified between Vehicle-CR and BCI-CR microbiota by ANCOM-BC2 analysis. Data are presented as the mean ± SEM. *N* = 7-8 mice of each group.

To investigate the DUSP6-mediated compositional changes in cold-adapted gut microbiota, we utilized microbial co-occurrence network analyses at the family level. We found that 2 days after the cold challenge, *Akkermansiaceae* dissociated from the major network and formed an isolated module with *Bacteroidaceae*, *Erysipelatoclostridiaceae*, etc. (Fig. 2a). In this initial cold-adapting stage, *Ruminococcaceae* formed the primary module with *Lachnospiraceae* and other *Clostridia* clades, which is similar to the structure of the *Ruminococcus* enterotype identified in humans^30^. After 7 days of cold exposure, *Akkermansiaceae* was eliminated from the network, and the major network was composed of high betweenness nodes (betweenness > 10) belonging to the *Clostridia* class, including the *Lachnospiraceae* and *Eubacterium coprostanoligenes* group (Fig. 2b). During the CR-transition, the edges of *A. muciniphila* were restored to those of high-betweenness families (colored nodes) for network recovery (Fig. 2c). In BCI-CR mice, the restoration of the ecological connections of *Akkermansiaceae* was abrogated by the inhibition of DUSP6, which contributed to the maintenance of a cold-like microbial module composed of *Ruminococcaceae, Lachnospiraceae* and other *Clostridia* clades (orange nodes) (Fig. 2d). By the co-occurrence analyses at the genus level, we observed that the prolonged DUSP6 inhibition led to the maintenance of the high-betweenness/low-degree centralities of the *Clostridia*/*Lachnospiraceae* clades during the CR transition (Supplementary Fig. 1j, k). This finding suggests that the DUSP6 inhibition could maintain the cold-adapted hierarchical structure of the gut microbiota in mice that underwent CR transition.

On the basis of these findings, we considered that the specific members of the *Lachnospiraceae* family may be critical for enhancing WAT browning during cold exposure. To identify potential browning-promoting bacteria in the cold microbiota, we performed an ANCOM-BC2 analysis on 16 S rRNA sequencing data from the BCI-CR and Vehicle-CR microbiota at the genus level. The results showed that during the cold-RT transition, the DUSP6 inhibition had a significant impact on maintaining the abundance of *Gordonnibacter*, UCG-10, *Monoglobus*, *Marvinbryantia*, *Eubacterium nodatum group*, and *Lachnospiraceae* uncultured genera (Fig. 2e). Notably, among these DUSP6-associated genera, only *Marvinbryantia* was significantly negatively correlated with the cold-induced changes in *Akkermansia* (Day 0 vs. Day 7) (Supplementary Fig. 1l). These findings indicate that *Marvinbryantia* could be a crucial genus influenced by DUSP6 regulation in response to cold conditions.

### Ursodeoxycholic acid contributes to the DUSP6-mediated remodeling of cold gut microbiota

Studies have shown that bile acids (BAs) play a role in regulating cold-induced thermogenesis by influencing the gut microbiota^20,31^. The most significant increases in bile acids in cold-exposed mice have been suggested to be cholic acid (CA), ursodeoxycholic acid (UDCA), and muricholic acid (MCA)^20^. To investigate whether *Dusp6* is involved in the regulation of gut microbiota remodeling in response to cold exposure through bile acids, the mRNA expression of major cytochrome P450 (CYP) enzymes in the liver tissues of WT and D6KO mice was analyzed via qRT-PCR analysis. We did not observe any significant difference between WT and *Dusp6-*deficient mice in the expression of hepatic *Cyp7a1*, *Cyp7b1*, *Cyp8b1*, *Cyp2c70* or *Cyp27a1* between WT and *Dusp6*-deficient mice (Supplementary Fig. 2a). Moreover, the expression of the hepatic *Dusp6* gene was also not altered by cold exposure (Fig. 1i). Furthermore, the BCI treatments did not significantly alter the expression dynamics of these CYP genes in the livers of mice that underwent CR transition (Supplementary Fig. 2b). These results suggest that *Dusp6* might not be involved in the regulation of host-derived BAs in mice subjected to cold stress.

**Fig. 3:**
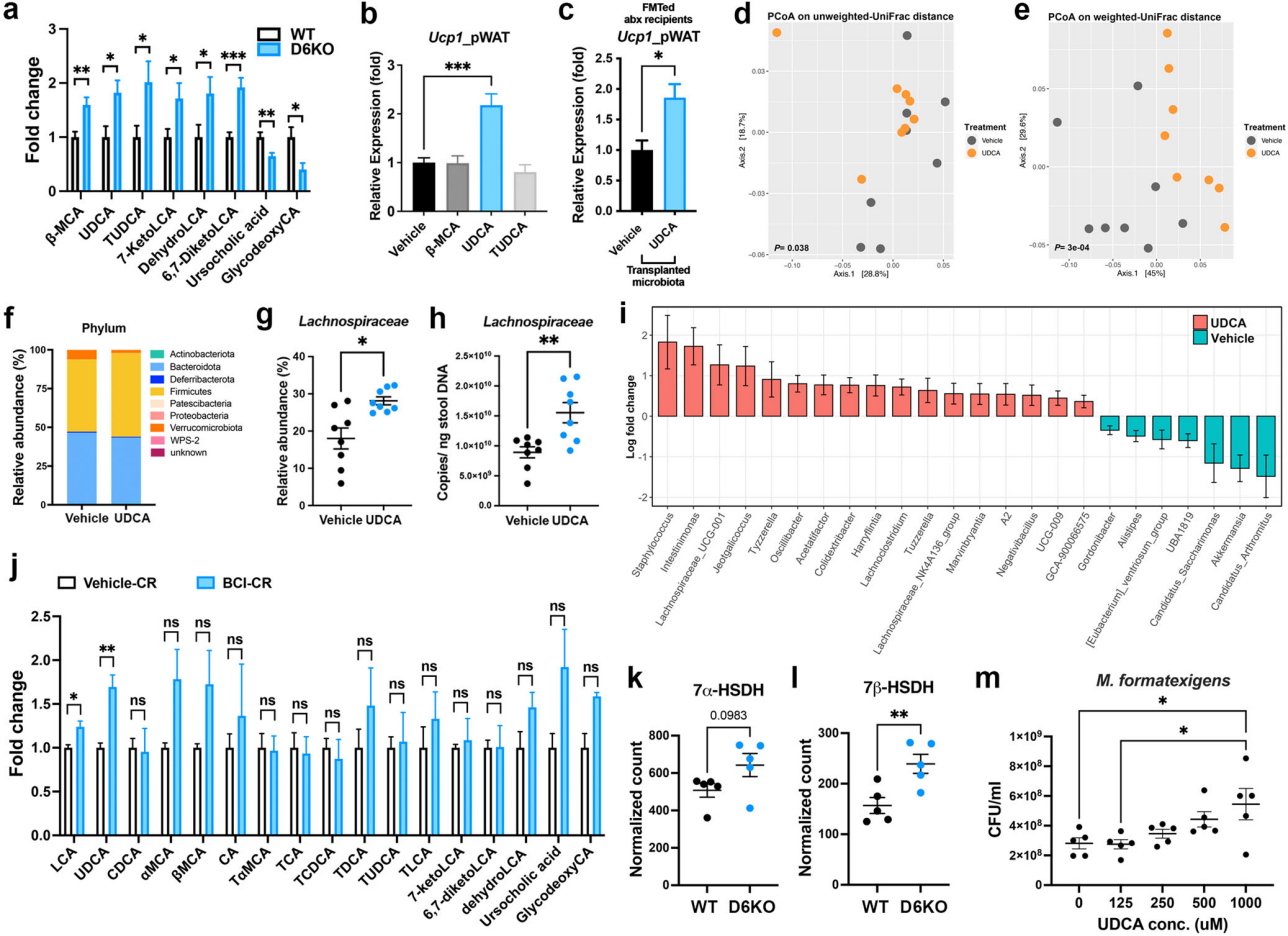
UDCA contributes to the *Lachnospiraceae* expansion in cold-adapted gut microbiota. **a** Relative fold changes of the significantly differential bile acids identified between the cecal content of WT and D6KO mice by LC-MS/MS analyses. *N* = 9-10 mice of each group. **b** qRT-PCR analysis of *Ucp1* expressions in pWAT of mice with a 7 days administration of vehicle, β-MCA, UDCA and TUDCA. *N* = 8 mice of each group. **c** qRT-PCR analysis of *Ucp1* expressions in pWAT of abx recipient mice transplanted with Vehicle- or UDCA-treated microbiota. *N* = 8 mice of each group. **d**, **e** PCoA plot based on unweighted (**d**) and weighted (**e**) UniFrac distance of gut microbiota of UDCA-administered mice. *N* = 8 mice of each group. *P-*value is calculated by pairwise PERMANOVA. **f** Microbial composition of Vehicle- or UDCA-treated microbiota at the phylum level. **g**, **h** Relative abundance (**g**) and numbers of 16S rRNA genes (**h**) of *Lachnospiraceae* in fecal DNA of Vehicle- and UDCA-treated mice. **i** The differentially enriched genera between Vehicle- and UDCA-treated microbiota were identified by ANCOM-BC2 analysis. **j** Relative fold changes of the UDCA-related bile acids were measured in cecal contents of Vehicle-CR and BCI-CR mice by LC-MS/MS analyses. *N* = 5 mice of each group. **k**, **l** Normalized counts of microbial 7α-HSDH and 7β-HSDH genes in gut metagenome of WT and D6KO mice. *N* = 5 mice of each group. **m** In vitro culture assays were conducted for *M. formatexigens* with the addition of varying doses of UDCA. *N* = 5 of each condition. **a**, **c**, **g**, **h**, **j**, **k**, **l** Data are presented as the mean ± SEM. *P < 0.05, **P < 0.01, ***P < 0.001 according to unpaired *t*-test. (**b**, **m**) Data are presented as the mean ± SEM. *P < 0.05, ***P < 0.001 according to One-Way ANOVA analysis and Tukey post-hoc test.

Interestingly, we observed that the abundance of certain cecal BAs, including β-muricholic acid (β-MCA), ursodeoxycholic acid (UDCA), tauroursodeoxycholic acid (TUDCA), dehydrolithocholic acid (dehydroLCA), 7-keto lithocholic acid (7-keto LCA) and 6,7-diketolithocholic acid (6,7-DiketoLCA), were significantly increased in *Dusp6-deficient* mice (Fig. 3a and Supplementary Fig. 2c). Because β-MCA, UDCA, and TUDC have been shown to be associated with the lipid metabolism in mice^32–34^, we further investigated whether the intestinal DUSP6 regulates the formation of browning-promoting gut microbiota through these three BAs. After a 7 day oral gavage treatment for the comparative purpose to the 7 day cold exposure, we found that only UDCA but not β-MCA and TUDCA showed a significant effect in inducing *Ucp1* expression in the pWAT of treated mice (Fig. 3b) while no significant difference in body weight was observed between the Vehicle- and UDCA-treated mice (Supplementary Fig. 2d). When transplanted UDCA-treated fecal microbiota (after a 7 day UDCA treatment) into abx mice, the UDCA-shaped microbiota indeed showed an enhanced browning-promoting ability compared with that of the vehicle-treated microbiota to increase *Ucp1* expression in pWAT without significantly changing body weights of transplanted mice (Fig. 3c and Supplementary Fig. 2e).

The administration of UDCA has been shown to specifically induce *Lachnospiraceae* expansion in mouse gut microbiota^35^. To confirm whether UDCA is a crucial effector that regulates the remodeling of cold-adapted microbiota, we performed 16 S rRNA sequencing on fecal DNA of vehicle- or UDCA-treated mice. After 7 days of treatment, we observed significant shifts in the gut microbiota of UDCA-treated mice based on unweighted- and weighted-UniFrac distances (Fig. 3d, e). Although the administration of UDCA had no significant impact on the total bacterial load (Supplementary Fig. 2f), it notably increased the alpha diversity (Supplementary Fig. 2g) within the gut microbiota as compared to that in the mice treated with the vehicle control. At the phylum level, we observed specific UDCA-mediated changes in Verrucomicrobiota and Firmicutes, while no significant alterations were detected in other major phyla of the mouse gut microbiota (Fig. 3f and Supplementary Fig. 2h–m). Notably, there was a significant increase in the relative abundance (Fig. 3g) and absolute 16 S rRNA copies (Fig. 3h) of *Lachnospiraceae* in the gut microbiota of UDCA-treated mice compared to vehicle-treated mice. The ANCOM-BC2 analysis further confirmed that the oral administration of UDCA effectively increased the abundance of certain *Lachnospiraceae* members, including *Lachnospiraceae* UCG–001, *Tyzzerella*, *Acetatifactor, Lachnoclostridium, Tuzzerella, Lachnospiraceae* NK4A136 group*, Marvinbryantia, Lachnospiraceae A2, GCA-900066575* (Fig. 3i). These results suggest that UDCA plays a critical role in promoting the expansion of *Lachnospiraceae* in mouse gut microbiota.

To further confirm that the DUSP6-UDCA axis plays a role in regulating cold-induced microbiota rearrangement, we used targeted LC‒MS/MS analysis to investigate the cecal BA profiles of vehicle-CR and BCI-CR mice. The results showed that the levels of UDCA and its metabolite LCA were significantly higher in mice that underwent CR transition after BCI treatment than after vehicle treatment (Fig. 3j). Although UDCA is considered as a secondary BA in humans, it has been suggested to be a primary BA in mice due to its occurrence in germ-free mice^36^. However, because the CONV-R mice have markedly greater levels of UDCA in the intestine, feces and serum than do germ-free mice, most UDCA is likely synthesized by the microbiota^37,38^. To address this point, we treated germ-free mice with BCI following the same procedure utilized for the BCI-CR mice. Subsequently, we conducted LC‒MS/MS analysis on cecal contents from BCI-treated germ-free mice. The results indicate that the inhibition of DUSP6 did not have an impact on the host-mediated synthesis of UDCA (Supplementary Fig. 2n). Furthermore, we performed shotgun metagenomic sequencing on microbial DNA extracted from the cecal contents of WT and D6KO mice. By specifically analyzing the levels of two major microbial genes involved in the biosynthesis of UDCA, including 7 alpha hydroxysteroid dehydrogenase (7α-HSDH) and 7 beta-hydroxysteroid dehydrogenase (7β-HSDH), we found that the D6KO microbiota possesses higher amount of both genes than did the WT microbiota; however, only the increase in 7β-HSDH is statistically significant (Fig. 3k, l). Within the *Lachnospiraceae* family, *Marvinbryantia* was the only genus that exhibited a significantly elevated abundance in both the BCI-CR (Fig. 2e) and UDCA-treated microbiota (Fig. 3i). In the *Marvinbryantia* genus (which belongs to the *Lachnospiraceae* family), most species are unculturable. One culturable human-origin member, *Marvinbryantia formatexigens*, has been selected as a member of a 14-species-composed synthetic microbiota (SM)^39^. Hence, we examined the potential of UDCA to promote the proliferation of *M. formatexigens*. Through an in vitro culture assay, we observed that UDCA dose-dependently enhanced the growth of *M. formatexigens* (Fig. 3m). Our results suggest that DUSP6 coordinates the production of microbial UDCA to regulate cold-induced gut microbiota remodeling and that *M. formatexigens* might be a critical mediator for promoting WAT browning.

### DUSP6 regulates the mucus-microbiota interface by altering the glycosylation of mucins

Because both *Akkermansiaceae* and *Lachnospiraceae* have been suggested to be enriched at the mucosal side of the intestinal epithelium^12^, we assessed whether the intestinal *Dusp6* can regulate the cold-associated changes in the gut microbiota by altering the host-microbe interface. By performing REACTOME enrichment analysis of the downregulated differentially expressed genes (DEGs with > 1.5-fold-change in D6KO/WT comparison and *p*-adj < 0.05) identified between ileums isolated from WT and D6KO mice, we noted that the two pathways related to the O-linked glycosylation of mucins were most significantly enriched (Supplementary Fig. 3a). These findings prompted us to investigate whether *Dusp6* regulates *A. muciniphila* abundance by altering the glycosylation of mucins.

*A. muciniphila* is a well-known mucin-degrading bacterium that could be a target hub for the host to control the composition of the gut microbiota through host-derived mucus^12^. When we evaluated the downregulated intestinal DEGs that are potentially involve in the glycosylation of mucins in D6KO mice, we found that the expression of *Dusp6* was positively correlated with the expression of genes regulating the fucosylation and O-linked glycosylation of mucins, including *Fut2*, *Galnt3*, *Galnt4*, *B3gnt7*, *Chst4*, *Hk2*, *Gfpt1*, *Muc4*, and *Muc13* (Supplementary Fig. 3b). Notably, *Galnt3*, *Galnt4*, and *B3gnt7* are important enzymes that regulate the attachment of N-acetylgalactosamine (GalNAc) and N-acetylglucosamine (GlcNAc) to mucins^40^. A recent study demonstrated that the regulation of epithelial fucosylation is crucial for shaping the gut microbiota composition^41^. Fucose, GlcNAc, and GalNAc are suggested to be the limited glycans that *A. muciniphila* can utilize^39^. We therefore hypothesized that *Dusp6* might affect the abundance of *A. muciniphila* through a host-feeding mechanism^12,13,42^. Lectin staining analyses indeed revealed that the colonic mucus of *Dusp6*-deficient mice exhibited reduced binding to wheat germ agglutinin (WGA, which binds specifically to GlcNAc) but not to *Vicia villosa* lectin (VVL, which binds preferentially to GalNAc) or *Ulex europaeus* agglutinin (UEA, which binds to fucose residues) (Supplementary Fig. 3c–f). In BCI-CR mice, both colonic and ileal mucus also showed reduced binding to WGA when compared with vehicle-CR mice (Supplementary Fig. 3g–i). Furthermore, we extracted colonic mucins from both WT and *Dusp6*-deficient mice and evaluated their ability to support the growth of *A. muciniphila*. These findings indicated that the colonic mucin proteins extracted from *Dusp6*-deficient mice exhibited a compromised ability to promote the in vitro growth of *A. muciniphila*, in contrast to the colonic mucin proteins extracted from WT mice (Supplementary Fig. 3j). These results indicate that *Dusp6* is involved in the host-mediated mechanism regulating cold-induced changes in the mucus-microbiota interface.

### *M. formatexigens* is an adipose-regulating gut bacterium

**Fig. 4:**
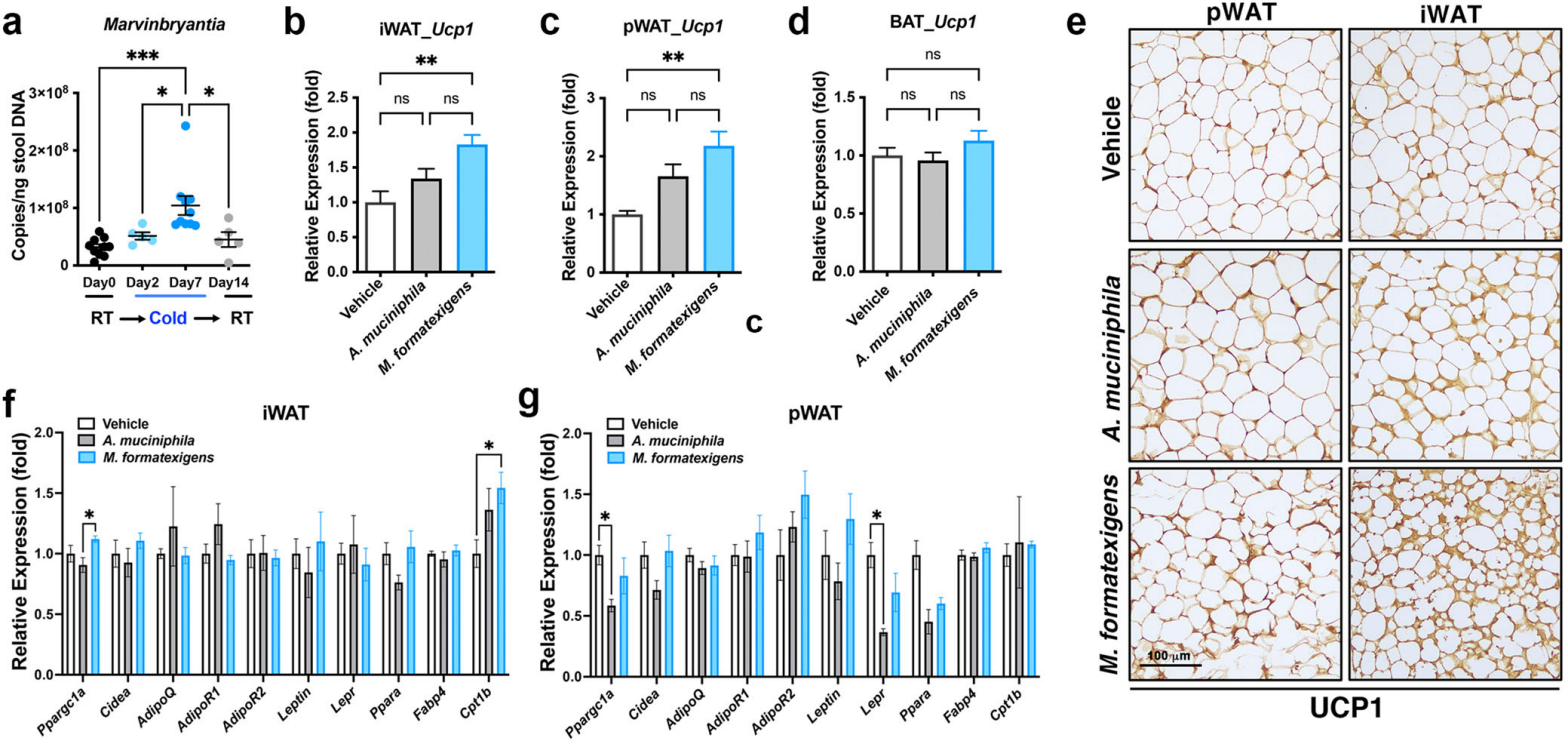
*M. formatexigens* is a browning-activating gut bacterium. **a** The absolute quantification of 16S rRNA gene copies of *Marvinbryantia* genus in the gut microbiota before (Day 0), during (Day 2 and Day 7) and after (Day 14) cold exposure. **b**–**d** qRT-PCR analysis of *Ucp1* mRNA expression in iWAT (**b**), pWAT (**c**) and BAT (**d**) of germ-free mice with a 3 days inoculation of vehicle (PBS), *A. muciniphila* (10^9^ CFU/day for 3 days) or *M. formatexigens* (10^9^ CFU/day for 3 days). *N* = 5 mice of each group. **e** Immunohistochemistry analysis of UCP1 expressions in pWAT and iWAT of germ-free mice with a 3 days inoculation of vehicle (PBS), *A. muciniphila* (10^9^ CFU/day for 3 days) or *M. formatexigens* (10^9^ CFU/day for 3 days). Scale bar, 100 μm. **f**, **g** qRT-PCR analysis of genes related to lipid metabolism in iWAT (**f**) and pWAT (**g**) of germ-free mice with a 3 days inoculation of vehicle (PBS), *A. muciniphila* (10^9^ CFU/day for 3 days) or *M. formatexigens* (10^9^ CFU/day for 3 days). *N* = 5 mice of each group. **a**, **b**, **c**, **d**, **b**, **g** Data are presented as the mean ± SEM. ns, non-significant; *P < 0.05; **P < 0.01; ***P < 0.001 according to One-Way ANOVA analysis and Tukey post-hoc test.

Our described results suggest that *M. formatexigens* might be a critical mediator that contributes to the regulation of cold-induced WAT browning. Supportingly, *Marvinbryantia* was confirmed to be a cold-induced genus in the cold microbiota by the absolute quantification of 16 S rRNA gene copies (Fig. 4a). To further confirm whether the WAT can rapidly monitor the browning cues derived from *M. formatexigens*, we conducted a short-term inoculation study in germ-free recipient mice using *A. muciniphila* or *M. formatexigens* (10^9^ CFU/mouse/day for 3 days). Our results showed that no significant difference in body weight was observed between the groups (Supplementary Fig. 4a). Notably, only *M. formatexigens* inoculation significantly enhanced *Ucp1* expression in both the iWAT and pWAT of inoculated germ-free mice and that germ-free mice inoculated with *A. muciniphila* did not significantly affect *Ucp1* expression (Fig. 4b, c). Moreover, neither *M. formatexigens* nor *A. muciniphila* significantly induced *Ucp1* expression in the BAT of inoculated germ-free mice (Fig. 4d). The *M. formatexigens*-mediated browning phenotypes in pWAT and iWAT were also confirmed by immunohistochemistry analyses on the UCP1 protein (Fig. 4e). In addition to *Ucp1*, we investigated whether *M. formatexigens* and *A. muciniphila* differentially regulate the expression of other lipid metabolism-related genes in the iWAT and pWAT of inoculated germ-free mice. The results showed that *M. formatexigens* significantly induced the expression of the beta-oxidation gene, *Cpt1b*, in iWAT but not in pWAT of inoculated germ-free mice (Fig. 4f, g). Differences in the ability of *M. formatexigens* and *A. muciniphila* to regulate the expression of *Ppargc1a* and *Lepr* in WAT were also observed (Fig. 4f, g). However, most lipid metabolism-related genes in the WAT of germ-free recipients were not significantly impacted by *M. formatexigens* or *A. muciniphila* inoculation. These results suggest that *M. formatexigens* is indeed a critical cold-associated gut bacterium that contributes to the regulation of white adipose browning.

### Nε-methyl-L-lysine is a microbial cue monitored by WAT

**Fig. 5:**
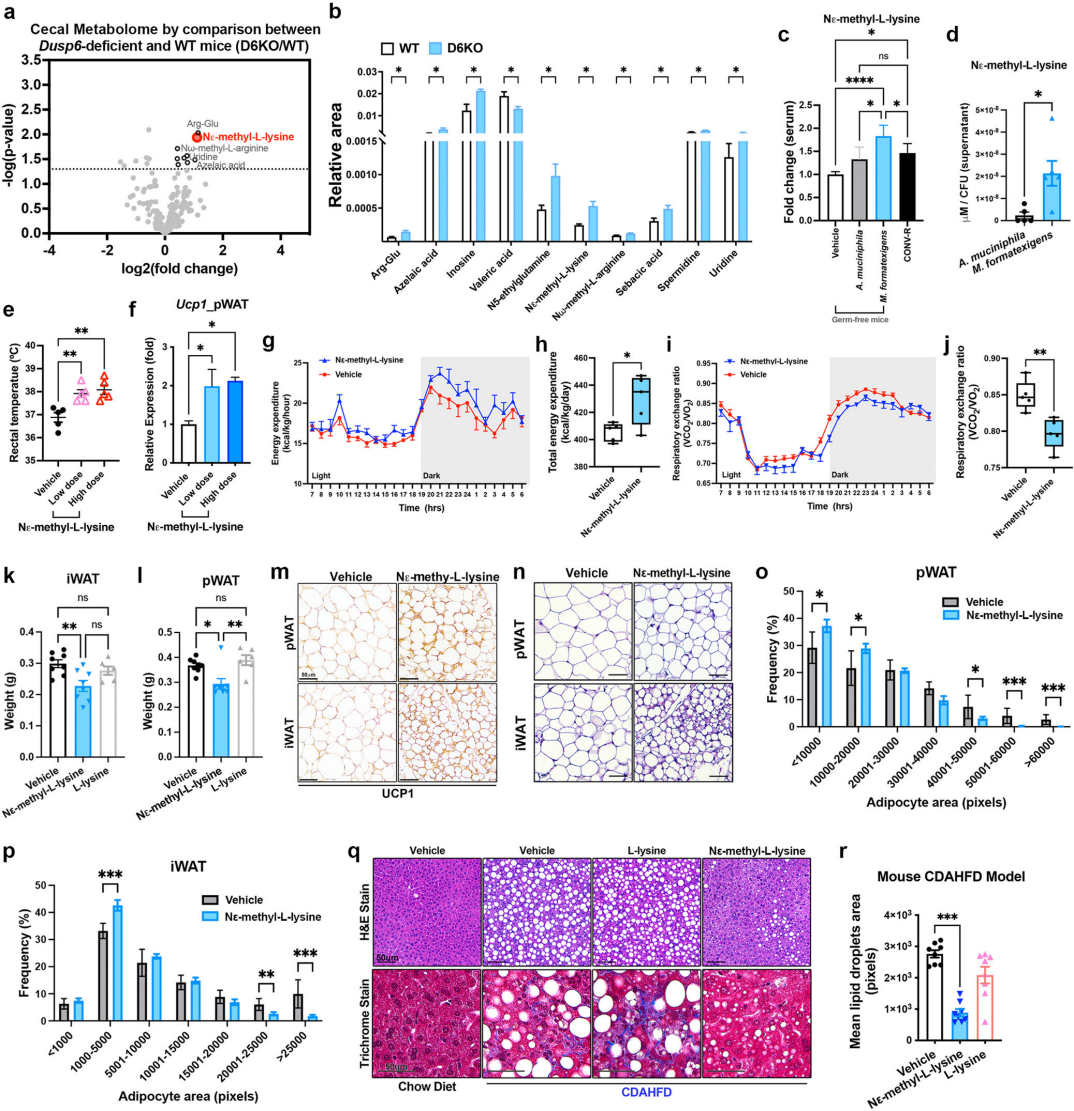
Nε-methyl-L-lysine is a microbial cue monitored by white adipose tissue. **a** Volcano plot of CE-MS metabolome analyses on cecal contents collected from D6KO or WT mice. The metabolites upregulated by *Dusp6* depletion are indicated by a black circle or red circle. *N* = 5 mice of each group. The cutoff of *P*-value is <0.05. **b** The upregulated cecal metabolites by *Dusp6* deficiency. *N* = 5 mice of each group. **c** Relative fold of serum Nε-methyl-L-lysine concentration of germ-free mice inoculated with *A. muciniphila* (10^9^ CFU/day for 3 days) or *M. formatexigens* (10^9^ CFU/day for 3 days), and CONV-R mice compared to the serum Nε-methyl-L-lysine concentration of vehicle (PBS)-treated germ-free mice. *N* = 3–5 of each group. **d** Nε-methyl-L-lysine in the cultural supernatant of *A. muciniphila* and *M. formatexigens* were quantified by LC-MS/MS analysis. *N* = 5 of each species. **e**, **f** Rectal temperature (**e**) and *Ucp1* mRNA expression in pWAT (**f**) of mice receiving a single administration of vehicle, and low (15 mM / 100 μl / mouse) or high (150 mM / 100 μl / mouse) dose of Nε-methyl-L-lysine. *N* = 5 for each group. **g**–**j** Daily total energy expenditure (**g**–**h**) and daily average respiratory exchange ratio (**i**, **j**) of vehicle- and Nε-methyl-L-lysine (150 mM / 100 μl / mouse)-treated mice; *N* = 5 for each group. **k**, **l** Total tissue mass of iWAT (**k**) and pWAT (**l**) in chow diet-fed male mice orally administrated Vehicle (H_2_O), Nε-methyl-L-lysine and L-lysine for 14 days. *N* = 6–8 mice of each group. (**m**, **n**) Representative images of UCP1 stained (**m**) or H&E stained (**n**) FFPE sections of pWAT and iWAT of chow-diet fed mice administrated Vehicle (H_2_O), and Nε-methyl-L-lysine for 14 days. Scale bars, 50 μm. **o**, **p** Analyses of adipocyte size distribution were performed by Adiposoft software on histological pWAT (**o**) and iWAT (**p**) sections stained with H&E of vehicle- and Nε-methyl-L-lysine treated mice. Data are presented as the mean ± SEM. *N* = 6–8 mice of each group. **q** Representative images of H&E-stained or Trichrome-stained FFPE sections of liver tissues collected from CD or CDAHFD-fed mice with the vehicle, L-lysine, or Nε-methyl-L-lysine administration. Scale bar, 50 µm. **r** Quantification of lipid droplets area was performed by Image J software on histological liver sections stained with H&E or masson’s trichrome stain of CDAHFD fed mice treated with the vehicle, L-lysine, and Nε-methyl-L-lysine. *N* = 8 mice of each group. (**b**, **d**, **h**, **j**, **o**, **p**) Data are presented as mean ± SEM. *P < 0.05; **P < 0.01; ***P < 0.001 according to unpaired *t*-test. (**c**, **e**, **f**, **k**, **l**, **r**) Data are presented as mean ± SEM. ns, statistically non-significant; *P < 0.05; **P < 0.01; ***P < 0.001; ****P < 0.0001 according to One-Way ANOVA analysis with Tukey post-hoc test.

Because our results suggest that the DUSP6-*M. formatexigens* axis may help identify the microbial cues regulating WAT browning, we performed untargeted CE-MS metabolome analyses of the cecal contents of WT littermates and *Dusp6*-deficient mice to identify potential browning activators. The results demonstrated that multiple cecal metabolites are upregulated by *Dusp6*-deficiency (Fig. 5a, b and Supplementary Fig. 4b). We chose ten commercially available metabolites, namely, azelaic acid, inosine, valeric acid, N5-ethylglutamine, Nε-methyl-L-lysine, Nω-methyl-L-arginine, sebacic acid, spermidine and uridine, to test their browning-promoting effects on differentiated mouse 3T3-L1 adipocytes. After 7 days of treatment postdifferentiation, we found that Nε-methyl-L-lysine was the only metabolite that could induce the expression of the *Ucp1* gene in differentiated 3T3-L1 adipocytes (Supplementary Fig. 4c). By LC–MS/MS analysis (Supplementary Fig. 4d), we found that CONV-R mice had significantly higher serum Nε-methyl-L-lysine levels than did germ-free mice (Fig. 5c). To confirm the role of the gut microbiota in influencing systemic Nε-methyl-L-lysine levels, we fasted CONV-R mice and GF mice for 48 h. The results showed significant lower serum Nε-methyl-L-lysine levels in fasted mice than in mice fed ad libitum (AL) (Supplementary Fig. 4e), such a reduction was not detected in fasted germ-free mice compared to GF mice under AL feeding (Supplementary Fig. 4f). The decline in serum Nε-methyl-L-lysine levels of fasted CONV-R mice may be linked to the reduction in the gut bacterial load (Supplementary Fig. 4g). These results suggest that gut microbiota plays a crucial role in modulating systemic Nε-methyl-L-lysine levels.

As the UDCA-*M. formatexigens* axis emerged as a crucial regulator of WAT browning, we compared the serum Nε-methyl-L-lysine levels in abx mice transplanted with microbiota treated with either vehicle or UDCA (Fig. 3c). The results indicated that the transplantation of UDCA-shaped microbiota led to significantly higher serum Nε-methyl-L-lysine levels in recipient mice than in mice that received vehicle-treated microbiota (Supplementary Fig. 4h). Furthermore, in the germ-free mice inoculated with *M. formatexigens* (10^9^ CFU/mouse/day for 3 days), there was a significant increase in the serum Nε-methyl-L-lysine concentration; in the mice inoculated with *A. muciniphila* (10^9^ CFU/mouse/day for 3 days), there was not a significant increase (Fig. 5c). We also observed significantly higher levels of Nε-methyl-L-lysine in the supernatant (Fig. 5d) and pellet (Supplementary Fig. 4i) of in vitro *M. formatexigens* cultures and lower Nε-methyl-L-lysine level in *A. muciniphila* cultures. These results suggest that Nε-methyl-L-lysine is an important microbial cue regulated by the UDCA-*M. formatexigens* axis.

To confirm that Nε-methyl-L-lysine is a microbial cue that can be detected in mouse WAT, we administered a single oral dose of Nε-methyl-L-lysine at a low (15 mM /100 µl / mouse) or high (150 mM /100 µl / mouse) dose to chow diet-fed mice. LC‒MS/MS analysis revealed that the mice administered a low dose of Nε-methyl-L-lysine had serum Nε-methyl-L-lysine levels similar to those of D6KO mice (Supplementary Fig. 4j). Twenty-four hours after a single administration, both low and high doses of Nε-methyl-L-lysine led to significant increases in the rectal temperature (Fig. 5e) and *Ucp1* expression in both the pWAT and iWAT but not BAT of treated mice (Fig. 5f and Supplementary Fig. 4k-l). Through indirect calorimetry, we observed a significant increase in 24 h energy expenditure (Fig. 5g, h) and a reduction in the respiratory exchange rate (Fig. 5i, j) in mice following a single high-dose administration of Nε-methyl-L-lysine. These results suggest that Nε-methyl-L-lysine can promote lipid utilization for energy.

To further examine whether supplementation with Nε-methyl-L-lysine can effectively reduce adiposity in mice, we administered Nε-methyl-L-lysine (150 mM /100 µl / mouse) or nonmethylated L-lysine (150 mM / 100 µl / mouse) to chow diet (CD)-fed mice for 14 days. The results demonstrated that Nε-methyl-L-lysine but not nonmethylated L-lysine significantly reduced the total mass of iWAT (Fig. 5k) and pWAT (Fig. 5l) and fasting glucose levels (Supplementary Fig. 4m) in treated mice without reducing body weight (Supplementary Fig. 4n). Nε-methyl-L-lysine also enhanced the expression of the *Ucp1* and *Cpt1* genes in pWAT, whereas non-methylated L-lysine did not (Supplementary Fig. 4o, p). The browning phenotype of pWAT and iWAT was also confirmed by immunohistochemistry analyses of the UCP1 protein (Fig. 5m). By histological examination, we found that the administration of Nε-methyl-L-lysine significantly reduced adipocyte size in both pWAT and iWAT (Fig. 5n). These results were confirmed by AdipoSoft software^43^, which showed that the size distribution of adipocytes in pWAT and iWAT was significantly reduced (Fig. 5o, p) and that the densities of adipocytes in pWAT and iWAT (Supplementary Fig. 4q, r) were significantly increased in Nε-methyl-L-lysine-treated mice. Our findings demonstrated that Nε-methyl-L-lysine is an effective microbiome-associated metabolite that reduces adiposity in mice by enhancing adipose browning.

Because we found that Nε-methyl-L-lysine can reduce pWAT mass in mice (Fig. 5l), we further assessed whether Nε-methyl-L-lysine has the potential to serve as a treatment for visceral fat-associated diseases. To this end, we used a choline-deficient, L-amino acid-defined, high-fat diet (CDAHFD) to induce nonalcoholic steatohepatitis (NASH) in mice (Supplementary Fig. 4s)^43^. Even though the liver-targeting CDAHFD mouse model can cause weight loss to interfere the observation of adipose functions (Supplementary Fig. 4t), we still observed that the high dose administration of Nε-methyl-L-lysine can significantly increase the expression of *Cidea*, but not *Ucp1*, in the pWAT of CDAHFD-fed mice (Supplementary Fig. 4u). No significant differences in pWAT mass or iWAT mass was observed between the various treatment groups under CDAHFD treatment (Supplementary Fig. 4v, w). Compared with CD-fed mice, CDAHFD-fed mice in the vehicle- and lysine-treated groups but not in the Nε-methyl-L-lysine-treated group exhibited significantly increased serum AST levels (Supplementary Fig. 4x). Nε-methyl-L-lysine significantly reduced liver lipid droplet (LD) density (Fig. 5q, r) and fibrosis (Fig. 5q and Supplementary Fig. 4y) in CDAHFD-fed mice. These findings suggest that Nε-methyl-L-lysine can alleviate liver damage caused by excessive lipid storage.

Given that browning primarily occurs in adipose tissues, we investigated whether Nε-methyl-L-lysine influenced the expression of relevant genes associated with fatty acid β-oxidation and gluconeogenesis to explore the potential mechanism through which Nε-methyl-L-lysine reduces hepatic lipid accumulation in CDAHFD-treated mice. We found that the expression of genes involved in β-oxidation and gluconeogenesis, such as *Cpt1b*, *Cpt2*, *Ehhadh*, *Hadha*, *Cpk1*, *Fbp1*, *G6pc*, *Gck*, and *Pcx*, did not significantly change in the livers of CDAHFD-treated mice after Nε-methyl-L-lysine treatment (Supplementary Fig. 4z). However, we observed a significant reduction in the expression of the LD-promoting gene *Plin2* (Supplementary Fig. 4z). PLIN2 is the most abundant perilipin protein in the liver and is highly correlated with LD abundance and nonalcoholic fatty liver disease (NAFLD) in humans^44^. Studies have demonstrated that the liver-specific ablation of PLIN2 can effectively decrease hepatic LD accumulation and prevent the development of NASH and fibrosis in mice^45^. However, further research is needed to better understand how Nε-methyl-L-lysine reduces *Plin2*-dependent hepatic lipid accumulation at the molecular level.

**Fig. 6:**
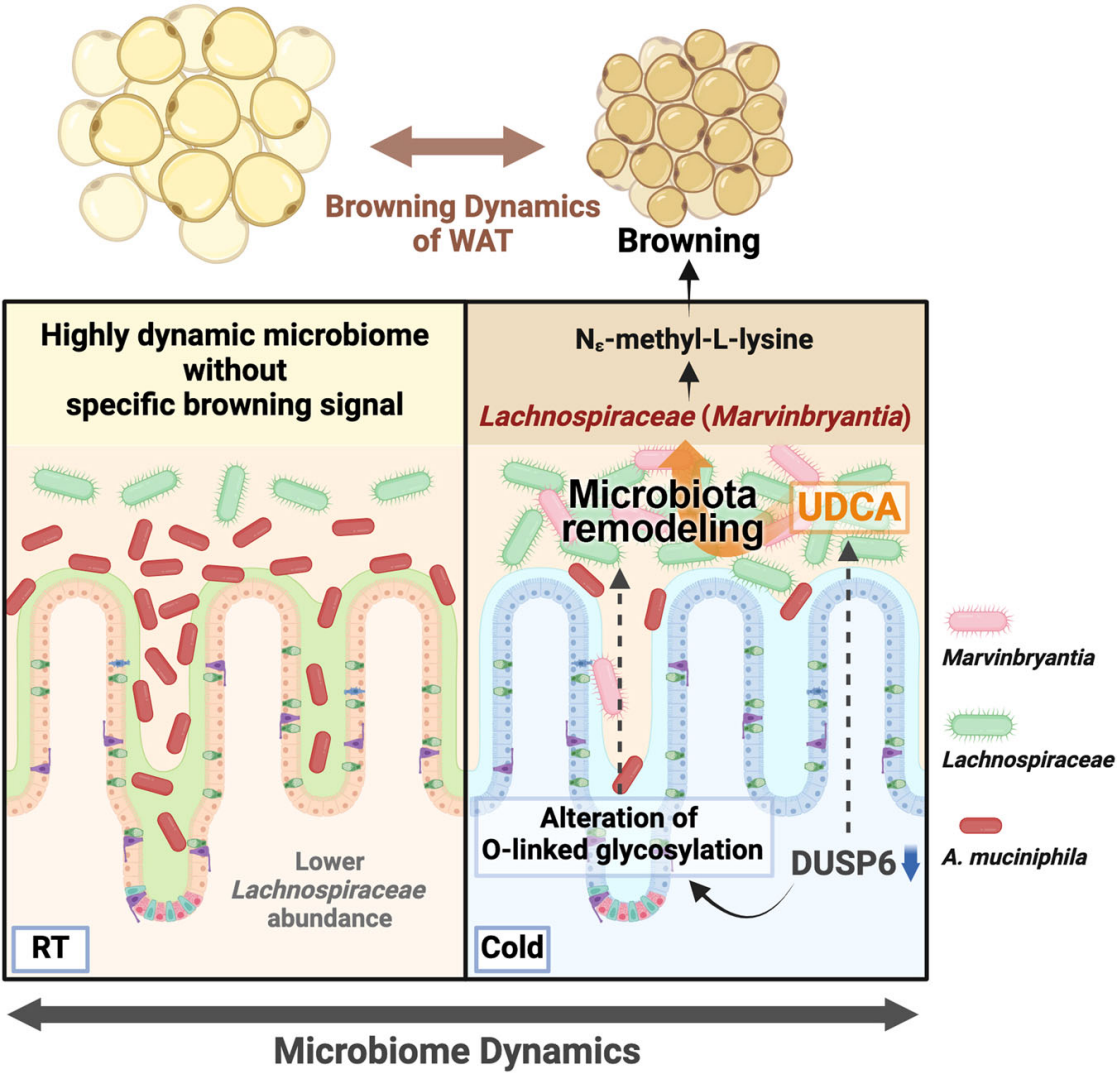
Graphical summary of the potential roles of DUSP6 in regulating microbiota remodeling. The decrease in intestinal DUSP6 expression due to cold exposure contributes to the expansion of *Lachnospiraceae* through a UDCA-dependent mechanism. The *Marvinbryantia* genus plays a critical role in promoting cold-induced browning in WAT.

Taken together, the findings of the present study revealed that the DUSP6-UDCA-*M. formatexigens* axis is a host-microbe coadaptation mechanism that regulates the remodeling of the cold microbiota (Fig. 6). Our results demonstrate that the dynamics of the gut microbiota are important for regulating specific molecular cues to increase host acclimation capacity^1^. Understanding the host control mechanism regulating specific rearrangements in the gut microbiota might facilitate the development of drugs for human diseases.

## Discussion

The mammalian gut metagenome plays a profound role in regulating phenotypic adaptations to environmental challenges^1,46^. Cold exposure has been shown to remodel the composition of the gut microbiota and promote WAT browning, which leads to fat loss in mice^19^. This finding strengthens the importance of microbiota rearrangements for regulating host physiology^1^. Accumulating evidence suggests that *Dusp6* deficiency has significant health benefits via a gut microbiome-dependent mechanism^28,47^. However, there is currently no approved therapy for depleting or inhibiting intestinal DUSP6 in humans. Therefore, identifying the specific conditions that can downregulate intestinal DUSP6 in CONV-R WT mice may help facilitate a better understanding of how a host gene can reversibly influence the composition of the gut microbiota. Through chemical screening in zebrafish, (E/Z)-BCI has been shown to be a promising compound that targets DUSP6^48^. However, to provide evidence that the effects of *(*E*/*Z)-BCI are due to specific DUSP6 inhibition, the most critical phenotypes observed in BCI-treated mice were also confirmed in *Dusp6*-deficient mice in this study. Additionally, we confirmed that *(*E*/*Z)-BCI*-*induced WAT browning was absent in D6KO mice, suggesting that the effects observed in this study primarily stemmed from a DUSP6-dependent mechanism. Investigating the complex three-way interactions among the environment, genetics, and gut microbiome is challenging. To gain insights into host-mediated microbiota modulation during the short CR window, we employed pharmacological inhibition of DUSP6. Although our findings indicated a relative specificity of decreased *Dusp6* expression in the intestine, it is possible that (E/Z)-BCI could have unknown effects on various organs. Therefore, the use of intestine-specific knockout animals could offer further clarity regarding the role of intestinal DUSP6 in influencing adipose functions.

A rearranged microbiota might change its function by altering metabolomic outputs^49^. However, an often-asked question in the microbiome field is whether the rearrangement of the gut microbiota can occur rapidly enough to counteract the speed of environmental changes and also be synchronously monitored by the host’s organs^1^. This study revealed that cold microbiota can adapt to cold exposure rapidly. Within 2 days following the transition from cold to room temperature, the gut microbiota lost its ability to stimulate WAT browning. To determine whether WAT can promptly detect such alterations in the gut microbiome, all FMT experiments were conducted only once to evaluate the sensitivity of WAT to rapid changes in the microbiota. Our results indicated that DUSP6-mediated microbiome dynamics could quickly contribute to browning signaling in WAT. Even after only a single FMT, the gut microbiota of BCI-treated mice undergoing cold-RT transitions can enhance *Ucp1* expression in the WAT of transplanted mice. Moreover, since the browning phenotypes in WAT differ significantly between obese and lean animals, our approach helps eliminate the possibility that these variations in browning signaling attribute to metabolic differences.

*A. muciniphila* is a critical beneficial bacterium for human health. However, it is still unclear whether the dynamics of *A. muciniphila* abundance contribute to the physiological rearrangement of the gut microbiota. Interestingly, specific rearrangement between *Akkermansiaceae* and *Lachnospiraceae* has been observed in patients receiving prolonged antibiotic treatment, suggesting a potential role of *A. muciniphila* in mediating these changes^50^. The downregulation of *A. muciniphila* abundance is a well-known cold-adapted phenotype in mice^19^. In this study, we found that *Dusp6* deficiency could alter the glycosylation status of intestinal mucins, which contributed to a reduction in *A. muciniphila* abundance in the mouse gut. Both *A. muciniphila* and *M. formatexigens* have been selected as members of a 14-species-composed synthetic microbiota (SM)^39^. In germ-free mice transplanted with this SM, *A. muciniphila* was shown to be negatively correlated with *M. formatexigens* under fiber-rich diet and fiber-free diet conditions^39^. In this study, we also noted an inverse relationship between *Akkermansia* and *Marvinbryantia*. It would be intriguing to explore whether the microbial competition between *A. muciniphila* and other gut commensals plays a role in regulating the physiological dynamics of the host.

Bile acids play a vital role in nutrient absorption and act as regulators in maintaining balanced lipid, glucose, and energy metabolism for overall metabolic equilibrium. Cold exposure can influence the conversion of cholesterol to bile acids, shaping the microbiota and regulating adaptive thermogenesis^20^. While our findings did not reveal significant effects of *Dusp6* deficiency or a DUSP6 inhibitor on the expression of major CYP enzymes responsible for primary bile acid synthesis, the noteworthy rearrangement of the microbiota in D6KO and BCI-treated mice contribute to alterations in the synthesis of UDCA-related bile acids. Notably, we indeed found that the D6KO microbiome contained more microbial 7α-HSDH and 7β-HSDH genes than did the WT microbiome. Genetic factors have profound impact on bile acid levels^51^. While UDCA was suggested to be the primary bile acid in mice, we verified that BCI did not induce significant changes in the levels of host-derived UDCA in germ-free mice. These results underscore the significant role of host genetics in shaping the composition of the microbiota to produce secondary bile acids. Our observations revealed that in cold microbiota, DUSP6 inhibition preserved the centrality of *Ruminococcacea*e and *Lachnospiraceae* within the microbial network. Given that both *Ruminococcaceae* and *Lachnospiraceae* abundances have been linked to UDCA levels^52^, further investigation is required to identify the specific UDCA producer regulated by DUSP6.

UDCA has been demonstrated to stimulate adipose browning by activating the SIRT-1-PGC1-α signaling pathway and reducing overall body adiposity^53^. Our study further addresses the role of microbiota-dependent mechanisms in UDCA-mediated regulation of adipose tissue functions. While we cannot completely rule out the direct influence of UDCA on WAT browning, mice transplanted with UDCA-shaped microbiota show an enhanced browning phenotype in WAT. Additionally, we observed that UDCA can promote the growth of the browning bacterium, *M. formatexigens*. Therefore, UDCA may have a coordinated impact on both adipocytes and gut microbiota, thereby regulating host metabolism.

Homeostasis of the amino acid pool is critical to human physiology and influences the growth of commensals^54^. The gut microbiota plays a profound role in maintaining the stable state of host nutrition through its de novo biosynthesis of amino acids^55^. Since dietary lysine levels are limited, recent studies have suggested that gut microbiota-derived amino acids are essential for maintaining systemic lysine levels^56^. A previous study demonstrated that Nε-methyl-L-lysine is present in bacterial flagellar proteins^57^. Free Nε-methyl-L-lysine has been detected in human plasma, and its concentrations are greater in fasted subjects than in nonfasted subjects^58^. To date, the biological sources and functions of free Nε-methyl-L-lysine have not been determined. In this study, we identified Nε-methyl-L-lysine as a microbiota-derived browning activator that has the notable ability to reduce adiposity in mice. Like lactate, which can be produced by both the host and microbiota, shifts in microbiota composition could influence host metabolism by altering microbiota-associated levels^59^.

Brown adipocytes represent a specialized cell type characterized by a high mitochondrial content and UCP1 expression, which enables them to engage in lipid burning for thermogenesis^60^. In contrast to brown adipocytes residing in BAT, the increased abundance of beige cells within WAT can arise through two potential mechanisms^60^. First, beige cells in WAT may originate from the de novo differentiation of WAT-resident progenitor cells. Second, beige cells in WAT may arise from the transformation of existing white adipocytes through a trans-differentiation mechanism. Both pathways may coexist, with different stimuli preferentially activating one over another. In this study, we observed that both *M. formatexigens* and Nε-methyl-L-lysine possess the capacity to induce white adipose browning without affecting *Ucp1* expression in BAT. These observations suggest that *M. formatexigens* and Nε-methyl-L-lysine may be involved in either the de novo differentiation or trans-differentiation of beige cells in WAT, rather than directly activating the thermogenesis pathway. Moreover, while our results indicated that the decrease in *Dusp6* expression is triggered by cold exposure in the mouse intestine, this reduction was not observed in WAT, liver, and BAT. Instead, BAT displayed an increase in *Dusp6* expression in response to cold exposure. This suggests that DUSP6 may play different roles in regulating thermogenic phenotypes of WAT and BAT. However, further investigation will be necessary to substantiate this hypothesis.

In *Dusp6* knockout mice, the gut microbiota is modulated relatively in a fixed state with a fixed function because genetic manipulation constantly shapes the microbiota^28^. Therefore, we were unable to investigate the impact of DUSP6-microbiota dynamics on host physiology in our previous study. In this study, we focused on physiological and dynamic states of gut microbiome, which are rare and extremely difficult to explore^61^. Furthermore, we provide evidence that host-to-microbe-to-host (*Dusp6*-to-*M. formatexigens*-to-WAT) biochemistry is essential for the host to regulate cold-induced WAT browning, emphasizing the importance of host-control and the hologenome theory in microbiome regulation^1,13,61^. The results of our study suggest that not only the abundance of beneficial microbes but also the dynamic regulation of microbiota rearrangements may be essential for human health.

## Methods

### Mice

Wild-type C57BL/6JNarl mice were provided by National Laboratory Animal Center (NLAC), NARLabs, Taiwan. Mice were housed with a 13 h light/11 h dark cycle (light from 7 A.M. to 8 P.M.) at 22˚C and free access to autoclaved food and water. Mice were raised in individually ventilated cages (IVC) under specific pathogen-free (SPF) conditions. Germ-free mice were kept in the IsoCage Bioexclusion System (Tecniplast) under sterile conditions. The germ-free condition was assessed through qRT-PCR analysis of bacterial DNA representation using the Femto Bacterial DNA Quantification kit (ZYMO RESEARCH). Mice were grouped by similar weight upon arrival and were housed in at least three and less than five per cage. Carbon dioxide inhalation was used for euthanasia at the end of each experiment described in this study, in accordance with the guidelines and regulations set by the animal center of the Medical College at National Cheng Kung University. All experiments were approved by the Institutional Animal Care and Use Committee (NCKU-IACUC-108135) at National Cheng-Kung University.

Cold treatment was performed on 8 week-old male C57BL/6 J mice. 6 °C (cold) exposures were performed by using a light- and humidity-controlled incubator with individually ventilated cages. To prolong the DUSP6 inhibition during CR transition, DMSO-dissolved (E/Z)-BCI hydrochloride (Merck) (5 μg / g body weight) and vehicle control (DMSO) were intraperitoneally injected into mice right before the mice returned to the RT condition once a day during CR period. The appropriate amount of DMSO-dissolved (E/Z)-BCI and vehicle were diluted into a PBS solution prior to injection to ensure the final DMSO concentration could be well tolerated (<5%), and immediately injected into mice^62^. All the mice in CR experiments were on ad libitum feeding. The identical treatment condition used for BCI was administered to *Dusp6*-deficient mice in to explore off-target effects of BCI on adipose browning.

To investigate the browning-promoting and microbiota-remodeling capabilities of bile acids, mice were orally gavaged with β-MCA (70 μg / g body weight), UDCA (70 μg / g body weight), TUDCA (70 μg / g body weight) and vehicle (PBS) once a day for 7 days. To investigate the browning-promoting capabilities of *A. muciniphila* and *M. formatexigens*, 100 ul PBS suspensions of *Akkermansia muciniphila* BAA-835 or *Marvinbryantia formatexigens* DSM 14469 were administered into 8 week-old male germ-free C57BL/6 J mice (10^9^ CFU/mouse/Day) by oral gavage for 3 days. To test the browning-activating abilities of Nε-methyl-L-lysine (Merck), 8 week-old male C57BL/6 J mice were orally administrated Nε-methyl-L-lysine (15 mM or 150 mM; 100 μl/mouse/Day) or vehicle (water; 100 μl/mouse/Day) for once. To test the WAT-reducing abilities of Nε-methyl-L-lysine (Merck), 8 week-old male C57BL/6 J mice were orally administrated of L-lysine (150 mM), Nε-methyl-L-lysine (150 mM) (100 μl/mouse/Day) or vehicle (water; 100 μl/mouse/Day) for 14 days. The rectal temperature of mice was measured by using a rectal thermometer (Kent Scientific Co., US). Fasting blood glucose levels were measured following a 16 h fast by collecting blood from the tail vein using an Accu-Chek Instant glucometer (Roche Diagnostics, Germany).

### Indirect calorimetry

Energy metabolism of male C57Bl/6 J mice (age 8 weeks; chow diet) with a single administration of vehicle (H_2_O) or Nε-methyl-L-lysine (15 mM / 100 μl / mouse) was measured by indirect calorimetry (Comprehensive Laboratory Animal Monitoring System, CLAMS; Columbus Instruments) with one mouse / chamber. Following a 24 hacclimatization period, the mice were orally administered the vehicle (water) or Nε-methyl-L-lysine via gavage. Oxygen consumption and carbon dioxide production rates were continuously monitored for 24 h after gavage using the CLAMS system. Throughout the experiment, the mice had ad libitum access to food and water.

### Mouse NASH model

Groups of 6 week-old male C57BL/6 J mice were fed in choline-deficient, L-amino acid-defined, high-fat diet (CDAHFD; #A06071302) purchased from Research Diets Inc. or chow diet for 4 weeks. After 2 weeks of CDAHFD treatment, mice were orally administrated L-lysine (150 mM), Nε-methyl-L-lysine (150 mM) (100 μl/mouse/Day) or vehicle (water; 100 μl/mouse/Day) for 14 days with continuing CDAHFD treatment. Bodyweight was measured weekly. After sacrifice, the serum of mice was collected and stored at −80 °C. The serum level of aspartate aminotransferase (AST) was measured with biochemistry slides for a chemistry analyzer (FUJI DRI-CHEM 4000i, Fujifilm Corporation).

### Fecal microbiota transplantation (FMT)

Before FMT, microbiota depletion in mice was achieved by supplying drinking water containing antibiotics for 3 weeks^19,63,64^. Sterile water with a mixture of 100 µg/ml neomycin, 50 µg/ml streptomycin, 100 U/ml penicillin, 50 µg/ml vancomycin, 100 µg/ml metronidazole, 1 mg/ml bacitracin, 125 μg/ml ciprofloxacin, 100 μg/ml ceftazidime and 170 μg/ml gentamycin were freshly prepared and changed every 2 days. For performing FMT, donor mice were placed in clean autoclaved cages, and the fresh feces were collected within 15 min. Feces were suspended (1 g feces / 10 ml PBS) and centrifuged to obtain the fecal slurry for FMT. 300 µl fecal slurry was individually administrated to age- and sex-matched microbiota-depleted (abx) mice by oral gavage^28^.

### DNA extraction, 16S rRNA sequencing and microbiota analysis

Mouse feces were directly excreted into DNA/RNA Shield buffer (Zymo Research) and stored at -20 °C. For absolute quantification, ZymoBIOMICS Spike-in Control I (Zymo Research) with two known species was mixed with each mouse fecal sample and then subjected to the DNA extraction following the protocol provided by the manufacturer (Zymo Research). PCR amplification was performed on extracted DNA for 16 S rDNA amplicons by using the primers targeting the V3-V4 region (319 F: 5ʹ-CCTACGGGNGGCWGCAG-3ʹ and 806 R: 5ʹ-GACTACHVGGGTATCTAATCC-3ʹ). 16S rRNA amplicons were sequenced on the Illumina MiSeq™ sequencing platform (Illumina). The raw FASTQ files were analyzed using the QIIME 2 platform^65^ (version: 2020.8). Paired-end sequences were initially demultiplexed using the q2-demux plugin followed by denoising using DADA2^66^ via the q2-dada2 plugin, with resulting amplicon sequence variants (ASVs). Taxonomy was assigned to ASVs with SILVA^67^ database (v138) using the q2‐feature‐classifier plugin with the classify-consensus-blast^68^ method and the parameters (identity = 0.97). Beta diversity analysis was performed using Principal Coordinates Analysis (PCoA) based on the weighted UniFrac or unweighted UniFrac distances among samples, and non-metric multidimensional scaling (NMDS) analysis based on Bray-Curtis distances. The Permutational multivariate analysis of variance (PERMANOVA)/Adonis test was conducted using vegan: Community Ecology Package (R package version 2.5–7; http://CRAN.R-project.org/package=vegan). For ANCOM-BC2 (v2.0.3) analysis, ASVs were considered differentially abundant using ANCOM-BC2 if *P* < 0.05^69^.

Co-occurrence network was built by using Spearman’s rank correlations with a coefficient > |0.6| and *P*-value < 0.05 as threshold. Node degree and betweenness centrality are analyzed by R package igraph^70^. The betweenness centrality was measured according to the network, and the node size represents the value. Only the node with a betweenness higher than the preset cutoff (≥10 in Fig. 2a–d) was colored by class level. The network was visualized by Cytoscape software. Correlations between specific genera identified by ANCOM-BC2 (*P* < 0.05) were based on Spearman correlation coefficients (Supplementary Fig. 1I).

### Shotgun metagenomic sequencing

The shotgun metagenomic sequencings of indicated fecal DNA samples were performed by using Illumina NovaSeq 6000 platform (2*151 bp), generating an average of 52.2 million paired-end raw reads per sample (range: 46.8–54.9 million paired-end reads). After quality trimming, an average of 51.4 million paired-end reads per sample were preserved (range: 46.2–54.1 million paired-end reads). The protein sequences of bacterial 7alpha-HSDH and 7beta-HSDH from NCBI protein sequence database were used for the alignment of sequencing reads (randomly selecting 33 million reads) for each sample by using DIAMOND (v2.0.15.153) algorithm^71^.

### RNA extraction and quantitation RT-PCR (qRT-PCR)

After sacrifice, the mouse ileum was dissected and flushed with ice-cold PBS. Due to that the DUSP6-microbiota dynamics exhibit rapid changes during cold-RT transition, the ileal epithelium was scraped off using a coverslip to promptly collect and preserve samples at each timepoint, ensuring the preservation of the dynamic phenotypes. Ileal epithelium and other tissues were preserved in Allprotect Tissue Reagent (Qiagen) and stored at -80˚C till RNA extraction. Total RNA of ileal epithelium and WAT was extracted by using RNeasy Plus Mini kits (Qiagen) or RNeasy Lipid Tissue kits (Qiagen), respectively, according to the manufacturer’s instructions. The concentration and quality of RNA were measured by NanoDrop spectrophotometer (Thermo Fisher Scientific). For qRT-PCR analysis, complementary DNA was obtained by using M-MLV Reverse Transcriptase (Promega Corporation) with oligo-dT primers. qRT-PCR was performed with SYBR-green master (Roche Diagnostics USA) on a MyGo Pro PCR system (IT-IS Life Science). The primers used in this study are listed in Supplementary Table 1.

### Protein extraction and immunoblot analysis

Upon collection, tissues were frozen immediately with liquid nitrogen and stored at -80˚C. For protein extraction, isolated ileal epithelium was subjected to ice-cold RIPA Lysis and Extraction Buffer (Thermo Fisher Scientific) containing proteinase inhibitor cocktails (Millipore) and homogenized by beads-beating using Vortex-Genie 2 mixer (Scientific Industries, Inc.) for 30 min at 4˚C. After centrifugation, the supernatant was collected for immunoblot analysis. The total protein concentration was determined using the BCA Protein Assay Kit II (BioVision) and the measurements were detected using the Epoch microplate spectrophotometer (BioTek). For each sample, 10 μg of extracted protein was used for electrophoresis. The primary antibody against DUSP6 (Dilution: 1:3000, Cat# ab76310, RRID: AB_1523517, Abcam) and Actin (Dilution: 1:5000, Cat# MAB1501, RRID:AB_2223041, Sigma-Aldrich) were used in this study. Protein marker (PM2510, SMOBIO Technology, Inc.) was employed to determine the size of target proteins. The luminescence intensity was detected by the ImageQuant LAS 4000 mini-imaging system (GE Healthcare Life Sciences). Quantification of proteins on immunoblots was performed with ImageJ software (RRID:SCR_003070, NIH). Original uncropped blots were provided in the Supplementary Information. All blots were processed in parallel and derive from the same experiment.

### Histology

Tissues were fixed in 4% neutral-buffered formalin (Sigma-Aldrich), paraffin-embedded, sectioned, and stained with hematoxylin and eosin (H&E) or subjected to immunohistochemistry analysis. For mucus staining, 5 μm Carnoy-fixed paraffin-embeded sections were deparaffinated and stained with alcian blue (pH 2.5) according to the manufacturer’s instructions (Abcam). For lectin staining, 5 μm Carnoy-fixed paraffin-embeded sections were deparaffinated and antigen-retrieved using citrate buffer. The binding of biotinylated WGA, VVL, and UEA (Vector Laboratories) to tissue sections was performed using the VECTASTAIN Elite ABC-HRP Kit following the manufacturer’s instructions (Vector Laboratories). After lectin staining, the glycans bound with WGA, VVL, or UEA in colon or ileum tissue sections were visualized by DAB development. The counterstain of the nucleus was performed by using a Nuclear Fast Red reagent (Abcam). The binding intensities of lectins were quantified (3 measurements per section/4–5 mice per condition) by using IHC profiler software^72^. For adipose tissue, the 5 µm FFPE sections of pWAT and iWAT were deparaffinated and stained with H&E or anti-UCP1 antibody (Cat# ab10983, RRID: AB_2241462, Abcam). The mean adipocyte area and adipocyte size distribution were determined by using Adiposoft software. For quantifying lipid droplets in liver tissues, the 5 µm FFPE liver sections were deparaffinated and stained with H&E. The mean lipid droplets area was determined by using Image J software. For hepatic fibrosis staining, the 5 µm FFPE liver sections were deparaffinated and stained with a Trichrome staining kit (Cat# ab150686, Abcam) according to the manufacturer’s instructions. The binding intensities of lectins were quantified (3 measurement per section / 4–5 mice per condition) by using IHC profiler software^72^.

### RNA Sequencing

Functional enrichment analysis was performed on the DAVID^73,74^ website (v6.8) based on the databases of the REACTOME^75^ pathway. The ileal transcriptome data of D6KO and WT mice were obtained from our previously published dataset (BioProject ID: PRJNA320922 (SRA: SRP074626)).

### Bile acids profiling

The bile acid profiling using LC-MS/MS platform was carried out by BIOTOOLS CO., LTD. Briefly, 20 mg of cecal contents were mixed with 1000 μL of an extraction buffer containing an internal standard mixture. After vortexing for 30 s, the samples were homogenized at 35 Hz for 4 min and then sonicated for 5 min in an ice-water bath. Subsequently, the samples were incubated for 1 h at −20 °C and centrifuged at 12000 rpm for 15 min at 4 °C. The resulting supernatant was used for bile acid analysis. Chromatographic separation was achieved using a Waters ACQUITY BEH C8 column (2.1 mm × 100 mm × 1.7 μm), and mass analysis was performed utilizing the Waters Xevo TQ-S system in positive-ion ESI mode.

### Metabolomics

After sacrifice, the mice’s cecal contents were collected and stored immediately at -80 °C. The extraction protocol and reagents were provided by Human Metabolome Technologies (HMT Inc.). Briefly, 30–50 mg cecal contents of each mouse was resuspended in extraction buffer containing internal standards and then centrifuged. The supernatant of samples was transferred into a prewashed ultrafiltration column (5 kDa, UltrafreeMC-PLHCC) and centrifuged at 9100 x g for 1 h at 4 °C. Cecal metabolome was profiled by using Capillary Electrophoresis Time-of-Flight Mass Spectrometry (CE-TOFMS) in two modes for cationic and anionic metabolites by HMT Inc. 328 metabolites were detected on the basis of HMT’s standard library. For targeted detection of Nε-methyl-L-lysine, the cultural supernatants and pellets of *Akkermansia muciniphila* and *Marvinbryantia formatexigens* were collected and lyophilized by freeze dryer. For the serum samples, 10 μL of mouse serum was mixed with 40 μL ice-cold 100% methanol. The mixture was vortexed for 30 s and incubated at −80 °C for 20 min. Following incubation, the mixture was centrifuged at 15,000 g, 4 °C for 30 min, and the supernatant was carefully collected and dried under the nitrogen stream. The resulting dry residues were reconstituted in 80 μL of a solution containing Acetonitrile and Water in a 1:1 ratio for subsequent analysis. An injection volume of 5 μL from each sample was subjected to analysis using the Agilent 1200 Series Gradient HPLC System coupled with the API 5000 LC-MS/MS System (Sciex, US). Chromatographic separation was achieved using an Intrada Amino Acid Column (Imtakt, JP). The mobile phase system consisted of two components: mobile phase A, comprising 100 mM Ammonium formate in Water, and mobile phase B, consisting of 100% acetonitrile. For detection and quantification, multiple reaction monitoring (MRM) analyses were conducted and analyzed utilizing the Analyst Software (Sciex, US).

### Cultivation of bacteria

*Akkermansia muciniphila* was cultured by brain-heart infusion (BHI) broth. *Marvinbryantia formatexigens* DSM 14469 was cultured by modified GAM broth supplemented with 10% Rumen fluid. All species were cultured at 37 °C in an anaerobic culture apparatus. To evaluate the impact of UDCA on promoting the growth of *M. formatexigens*, we inoculated 10^6^ colony-forming units (CFU) of *M. formatexigens* into 1 ml of culture media with supplementation of a vehicle (DMSO) or varying quantities of UDCA. After a 24-hour incubation period, the CFU was quantified using the plating and counting method. For metabolomic analysis, bacterial culture was separated into supernatant and pellet by centrifugation. Metabolites in cultural supernatant and beads-bashed pellet were extracted by methanol-water buffer (1:1). The extracted metabolites of each sample were lyophilized by freeze dryer (FTS Systems Corrosion Resistant Freeze Dryer). The lyophilized residues were reconstituted in Acetonitrile/Water (1:1) buffer for LC-MS/MS analyses to detect Nε-methyl-L-lysine.

### Mucin extraction and the in vitro cultivation assay of *A. muciniphila*

The mucin extraction was performed by following the procedure described in previous studies^76–78^. Briefly, mucus was scraped from the mouse colon and lysed using a guanidium chloride extraction buffer (6 M GuHCl, 0.1 M Tris pH 8.0, 1 mM EDTA) supplemented with 2x protease inhibitor. The samples were gently homogenized and left to extract overnight at 4 °C on a rotator. Subsequently, the mixture underwent centrifugation at 20,000 g for 40 min at 4 °C, and the resulting supernatant was collected. To ensure comprehensive extraction, the lysate was successively redissolved in the guanidium chloride extraction buffer five times. All the extracted supernatants were combined to form a pooled extract of each mouse. The pooled extracts were then subjected to reduction using 100 mM dithiothreitol (DTT) for 5 h at 37 °C. An additional 100 mM DTT was added and allowed to react overnight at 37 °C. After reduction, the mucins were alkylated by 125 mM iodoacetamide overnight in the dark at room temperature. The purified mucins were dialyzed against ddH_2_O to remove unwanted components for further in vitro culture assay of *A. muciniphila*. To compare the impact of glycosylated mucins extracted from WT and D6KO mice on supporting the in vitro growth of *A. muciniphila*, the bacterial strain *Akkermansia muciniphila* BAA-835 was inoculated into BHI broth at a concentration of 1 × 10^6^ CFU / ml. Subsequently, the pasteurized mucins were introduced into the culture, resulting in a final concentration of 0.1 mg/ml. After 48 h cultivation, the culture was subjected to serial dilution and plated on BHI agar to enumerate the colony-forming units (CFU).

### Cell culture

3T3-L1 murine pre-adipocytes (Cat# SP-L1-F, RRID:CVCL 0123, ZenBio) were maintained following the protocol provided by the manufacturer. The differentiation of 3T3-L1 cells was performed by using 3T3-L1 DIFFERENTIATION KIT (DIF001, Merck). After differentiation, cells were maintained in a complete medium containing 50 μM azelaic acid, inosine, valeric acid, N5-ethylglutamine, Nε-methyl-L-lysine, Nω-methyl-L-arginine, sebacic acid, spermidine, uridine (all purchased from Merck) and vehicle control for 7 days before the harvest. After treatments, cells were subjected to RNA extraction and qRT-PCR analyses.

### Statistical analysis

Statistical analysis, excluding RNA-seq, was performed using Prism 5.0 (GraphPad Software). Comparisons for experiments between 2 groups were analyzed using an unpaired *t*-test. Any comparisons of >2 data sets were performed with an analysis of One-Way ANOVA followed by Tukey post-hoc test. Differences were considered significant when *P* < 0.05.

### Reporting summary

Further information on research design is available in the Nature Research Reporting Summary linked to this article.

### Supplementary information

Supplementary Information

Reporting summary

## Data Availability

The raw data of 16 S rDNA sequencing and shotgun metagenomic sequencing have been deposited to NCBI under the following BioProject ID: PRJNA798518. Any additional information required to reanalyze the data reported in this paper is available from the lead contact upon request.

## Code availability

This study does not report the original code. The detailed information of bioinformatic tools and software used in this study can be found in the Methods section.
