# Supplementary material for: Intestinal dual-specificity phosphatase 6 regulates the cold-induced gut microbiota remodeling to promote white adipose browning
Source: NPJ Biofilms Microbiomes. 2024 Mar 13;10:22. doi: 10.1038/s41522-024-00495-8 (PMC10937957; doi:10.1038/s41522-024-00495-8)

Supplementary Information

Intestinal Dual-specificity Phosphatase 6 Regulates the Cold-induced Gut Microbiota Remodeling to Promote White Adipose Browning

Pei-Chen Chen[#], Tzu-Pei Tsai[#], Yi-Chu Liao, Yu-Chieh Liao, Hung-Wei Cheng, Yi-Hsiu Weng, Chiao-Mei Lin, Cheng-Yuan Kao, Chih-Cheng Tai, Jhen-Wei Ruan*

[#] Equal contribution

* Corresponding author. Email: jhenweiruan@mail.ncku.edu.tw

Supplementary Figures

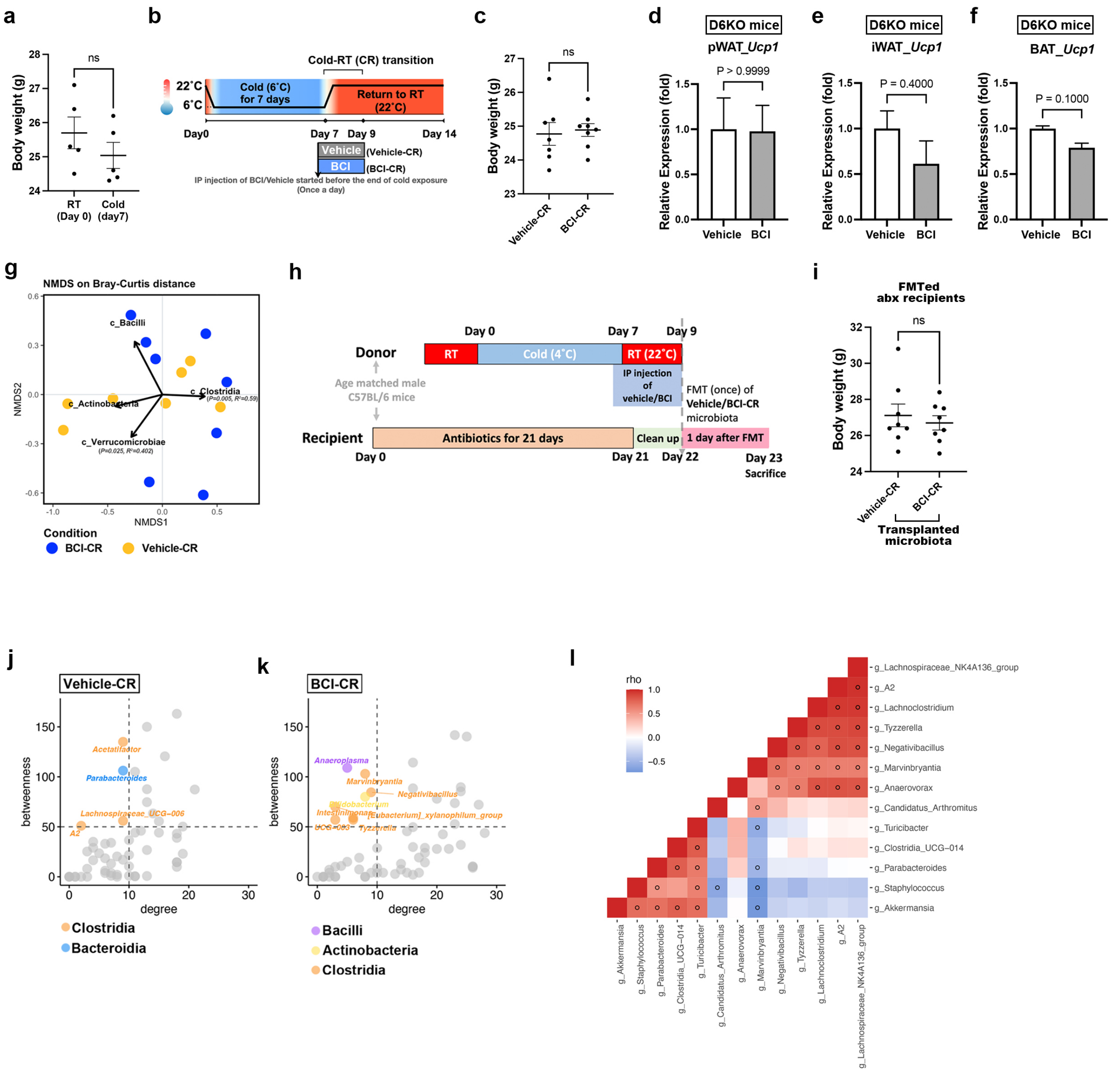

Supplementary Fig. 1. DUSP6 inhibition retains the cold microbiota composition during CR transition. (a) Body weights of mice with (7 days) or without (RT) cold exposure. N = 5 for each group. (b) The experimental scheme of BCI treatment during Cold-RT (BCI-CR) transition. CONV-R male C57BL/6 mice were exposed to cold environments (6˚C) for 7 days and intraperitoneally injected with vehicle (Vehicle-CR) or BCI (BCI-CR) once a day right before the termination of cold exposure for 7 days (Day 7 to Day 14). (c) Body weights of Vehicle-CR and BCI-CR mice. N = 7-8 for each group. (d-f) After a 3-day treatment of BCI on *Dusp6*-deficient (D6KO) mice via intraperitoneal injection, the mRNA expression of *Ucp1* was verified in pWAT (d), iWAT (e) and BAT (f) by qRT-PCR analysis. N = 3 for each group. (g) NMDS ordination based on Bray-Curtis indices. The arrows represent the significant variables (bacterial classes) contributing to the ordination of the microbiota in Vehicle-CR/BCI-CR comparisons (EnvFit analysis by permutation test, P < 0.05). (h) The experimental scheme of FMT of vehicle-CR and BCI-CR microbiota into abx mice. (i) Body weights of abx mice receiving single FMT of vehicle-CR or BCI-CR microbiota. N = 7-8 for each group. (j-k) The scatter plot of betweenness centrality and degree centrality of the vehicle-CR (j) and BCI-CR (k) microbiota in co-occurrence network at the genus level. The co-occurrence analyses were performed based on Spearman's correlation coefficient above 0.6 at a 0.05 P value significance. Hub genera with high betweenness centrality (≥ 50) and low node degree (≤ 10) in networks were colored by class. Each dot represents a genus. (l) Spearman correlation analysis was conducted on 13 genera that exhibited differential abundance as identified by ANCOM-BC2 (P < 0.05) in the RT/cold (Day 0 vs. Day 7) comparison. Significant correlations between any two genera (P < 0.05) are denoted by a black circle. (a, c, d, e, f, i) Data are presented as mean ± SEM. ns, statistically non-significant according to unpaired t-test.

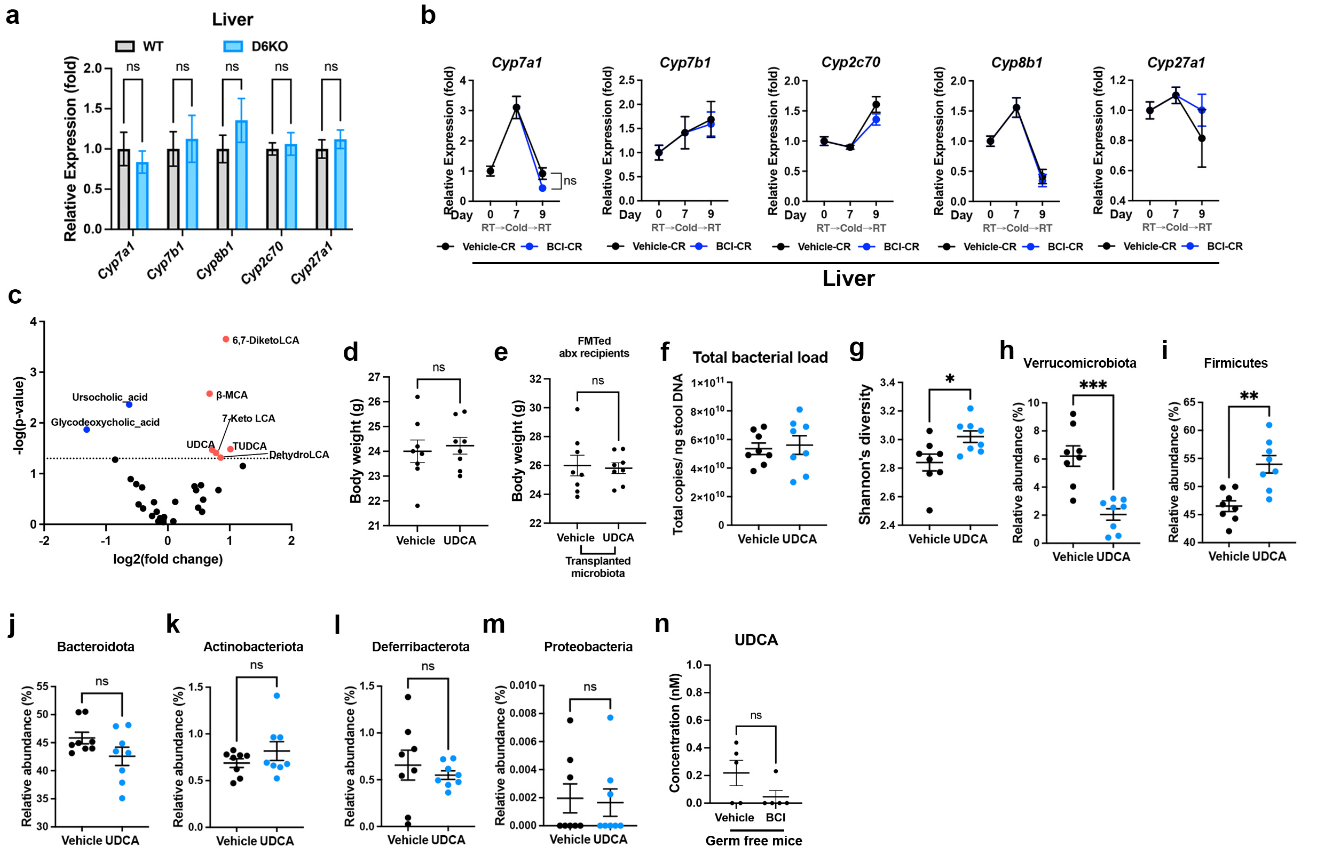

Supplementary Fig. 2. Hepatic *Dusp6* and CYP gene expression in mice exposed to cold stress. (a) qRT-PCR analysis of CYP genes mRNA expression in the liver of WT and D6KO mice. N = 10 mice for each group. (b) qRT-PCR analysis of CYP genes mRNA expression in the liver of mice that underwent CR transition. N = 5 mice in Cold-Day 0 and Day 7, N = 7-8 mice in Day 9 (Vehicle-CR and BCI-CR). (c) Volcano plot of the differential bile acids in cecal contents between D6KO and WT mice. (d) Body weights of mice receiving 7 days of Vehicle or UDCA treatments. (e) Body weights of abx mice receiving single FMT of vehicle- or UDCA-shaped microbiota. (f) Total bacterial 16S rRNA copies (per ng) in fecal DNA of Vehicle- and UDCA-treated mice. (g) Shannon's index of Vehicle- and UDCA-treated microbiota. (h-m) Relative abundance of Verrucomicrobiota (h), Firmicutes (i), Bacteroidota (j), Actinobacteriota (k), Deferribacterota (l) and Proteobacteria (m) phylum in gut microbiota of vehicle- or UDCA-treated (7 days) mice. (n) The serum levels of UDCA concentration in germ-free mice following a 3-day BCI treatment administered via intraperitoneal injection. (a, d, e, f, g, h, i, j, k, l, m, n) Data are presented as mean ± SEM. ns, statistically non-significant; *P < 0.05; **P < 0.01; ***P < 0.001 according to unpaired t-test. (b) Data are presented as mean ± SEM. ns, statistically non-significant according to One-Way ANOVA analysis and Tukey post-hoc test.

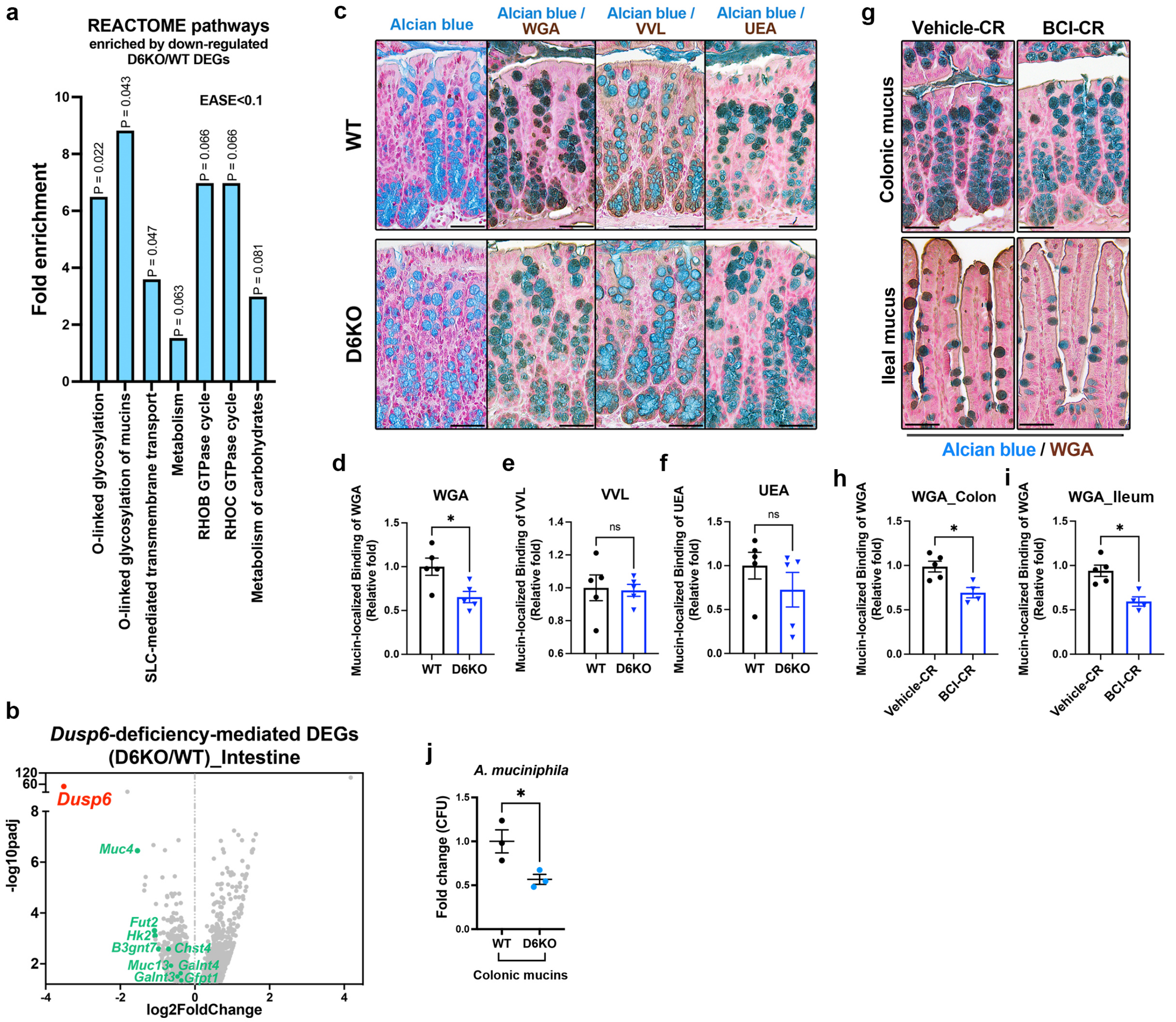

Supplementary Fig. 3. *Dusp6* regulates O-linked glycosylation of intestinal mucins. (a) Enrichment analysis of REACTOME pathways by intestinal DEGs downregulated by *Dusp6* deficiency. (b) Volcano plot of the distribution of DEGs in small intestines between D6KO and WT mice. The DEGs associated with the regulation in glycosylation of mucins were colored in green. (c) Alcian blue staining and immunohistochemistry of WGA (binds to GlcNAc), VVL (binds to GalNAc), and UEA (binds to fucose) were performed on the sections of formalin-fixed paraffin-embedded (FFPE) colon samples collected from WT and D6KO mice. Scale bar, 50μm. (d-f) Quantifications of WGA (d), VVL (e), and UEA (f) bindings were performed by ImageJ software on colon sections of 5 mice in each indicated group. (g) Alcian blue staining and immunohistochemistry of WGA were performed on the sections of FFPE colon and ileum samples collected from vehicle-CR and BCI-CR mice. Scale bar, 50μm. (h-i) Quantification of colonic (h) and ileal (i) WGA bindings were performed by ImageJ software on colon sections of 4-5 mice in each indicated group. (j) In vitro culture analysis of *A. muciniphila* with the supplementation of purified colonic mucins isolated from WT or D6KO mice in culture media. N = 3 mice for each group. (d, e, f, h, i, j) Data are presented as the mean ± SEM. ns, non-significant; *P < 0.05 according to unpaired t-test.

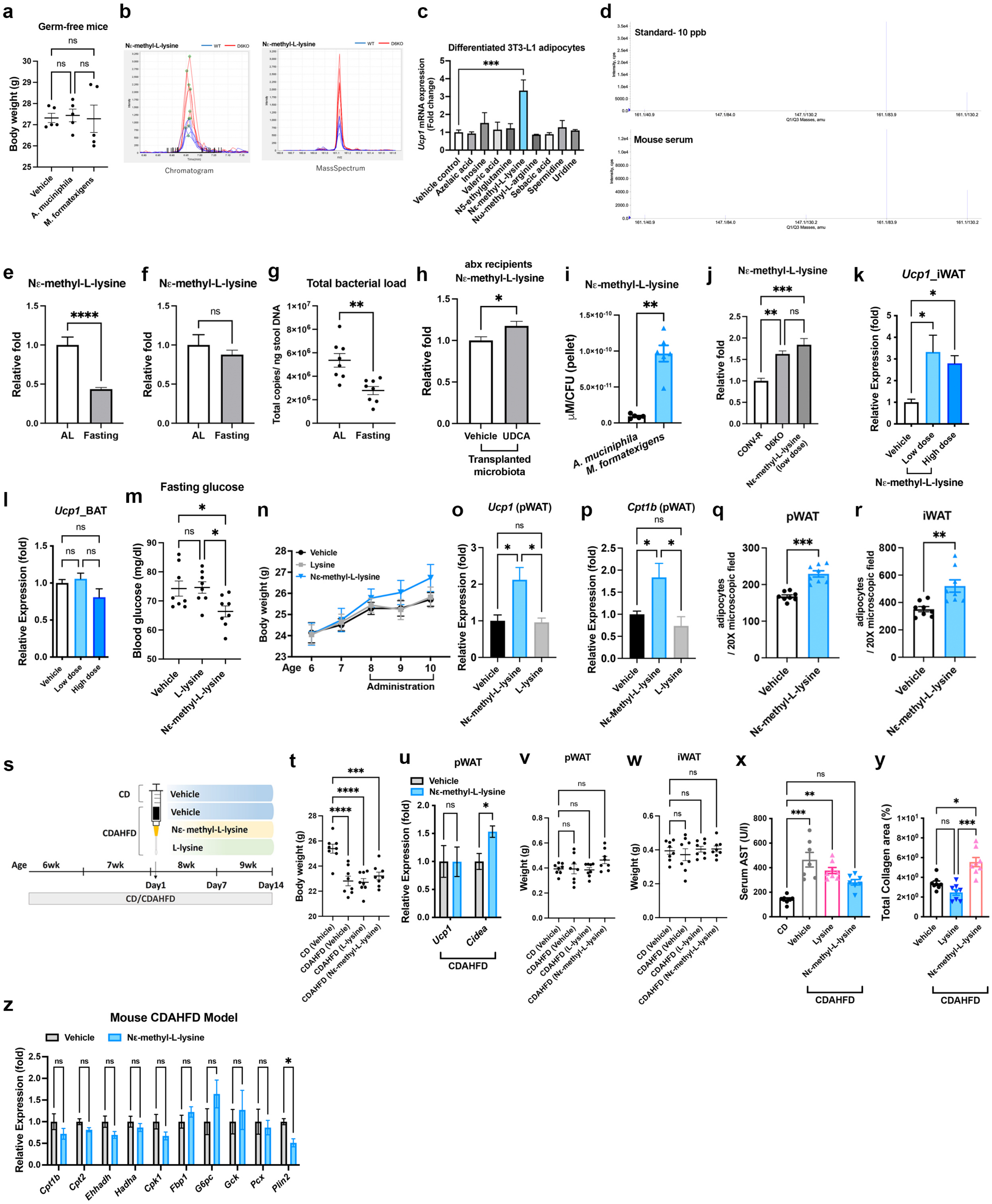

Supplementary Fig. 4. *Dusp6* regulates WAT browning in a microbiome-metabolomics-dependent manner. (a) Body weights of germ-free mice inoculated with vehicle (PBS), *A. muciniphila* (10^9 CFU / day for 3 days) or *M. formatexigens* (10^9 CFU / day for 3 days). N = 5 mice of each group. (b) Chromatogram and Mass Spectrum for Nε-methyl-L-lysine in untargeted CE-MS analysis described Fig. 5b. (c) qPCR analyses of *Ucp1* mRNA expression in differentiated 3T3-L1 adipocytes treated with 50μM indicated metabolite in complete medium. N = 3 of each metabolite. (d) Fragmentation patterns of Nε-methyl-L-lysine standard (04685, Sigma-Aldrich) and mouse serum Nε-methyl-L-lysine in LC-MS/MS analyses. (e-f) Fold changes in serum Nε-methyl-L-lysine levels of CONV-R (e) or germ-free (f) mice with or without a 48-hr fasting treatment. N = 5-8 mice of each group in (e) and N = 3 of each group in (f). (g) Total bacterial 16S rRNA copies (per ng) in fecal DNA of AL and fasted CONV-R mice. (h) Fold changes in serum Nε-methyl-L-lysine levels of abx mice receiving a single FMT of vehicle- or UDCA-shaped microbiota. N = 7 of each group. (i) Nε-methyl-L-lysine in the cultural pellets of *A. muciniphila* and

M. formatexigens were extracted for LC-MS/MS analysis. N = 5-6 of each species. (j) Fold changes in serum Nε-methyl-L-lysine levels of CONV-R mice, D6KO mice and mice receiving a single administration of low (15 mM / 100 μl / mouse) dose of Nε-methyl-L-lysine. N = 5 of each group. (k-l) *Ucp1* mRNA expression in iWAT (k) or BAT (l) of mice receiving a single administration of vehicle (PBS), and low (15 mM / 100 μl / mouse) or high (150 mM / 100 μl / mouse) dose of Nε-methyl-L-lysine. N = 5 for each group. (m) The blood glucose levels of mice receiving a 14-days administration of vehicle (PBS), L-lysine (150 mM / 100 μl / mouse) or Nε-methyl-L-lysine (150 mM / 100 μl / mouse) following a 16-hour fasting. N = 8 for each group. (n) Body weights of chow-diet-fed male mice orally administrated vehicle (H_2O, black), L-lysine (grey) and Nε-methyl-L-lysine (blue) for 14 days. N = 6-8 mice of each group. (o-p) qRT-PCR analyses of *Ucp1* (o) and *Cpt1b* (p) mRNA expression in pWAT of mice in (n). N = 6-8 mice of each group. (q-r) Analyses of adipocyte density were performed by Adiposoft software on histological pWAT and iWAT sections stained with H&E of vehicle- and Nε-methyl-L-lysine treated mice. (s) The experimental scheme of mouse CDAHFD-induced NASH model. (t-x) The body weights (t), *Ucp1* and *Cidea* mRNA expression in pWAT (u), pWAT mass (v), iWAT mass (w) and serum AST level (U/l) (x) of vehicle, L-Lysine and Nε-methyl-L-lysine treated mice fed with CD or CDAHFD. N = 7-8 mice of each group. (y) Quantification of fibrotic collagen area was performed by ImageJ software on histological liver sections stained with masson's trichrome stain of CDAHFD fed mice treated with the vehicle, L-lysine, and Nε-methyl-L-lysine. N = 8 mice of each group. (z) The mRNA expression of genes related to lipid metabolism and gluconeogenesis of vehicle and Nε-methyl-L-lysine treated mice fed with CDAHFD. N = 7-8 mice of each group. (e, f, g, h, i, q, r, u, z) Data are presented as mean ± SEM. ns, statistically non-significant; *P < 0.05; **P < 0.01; ***P < 0.001; ****P < 0.0001 according to unpaired t-test. (a, c, j, k, l, m, o, p, t, v, w, x, y) Data are presented as mean ± SEM. ns, statistically non-significant; *P < 0.05; **P < 0.01; ***P < 0.001 according to One-Way ANOVA analysis and Tukey post-hoc test.

Supplementary Table 1. Primer sets for qRT-PCR analyses

Gene	Primer sequences
Mouse gene	
Tbp-F	CAAACCCAGAATTGTTCTCC
Tbp-R	ATGTGGTCTTCCTGAATCCCT
Dusp6-F	TCCTAGGGACTGTGAGCAAACC
Dusp6-R	GGATCCAACCCGGTGTTTC
Hprt-F	TACTAGGCAGATGGCCACAG
Hprt-R	GCCTAAGATGAGCGCAAGTTG
Ucp1-F	GTGAACCCGACAACTTCCGAA
Ucp1-R	TGCCAGGCAAGCTGAAACTC
Cidea-F	TTCAAGGCCGTGTTAAGGA
Cidea-R	CCTTTGGTGCTAGGCTTGG
Cpt1b-F	TGAGCCAAACACCACGTTGCCA
Cpt1b-R	TAGAGCTCCACGTCATCGGCCA
Ywhaz-F	CCAGACTGAGGAAGATTAAGCAAT
Ywhaz-R	CAGTTCCAGGTATCATTTGTAATTT
Cyp7a1-F	AGCAACTAAACAACCTGCCAGTACT A
Cyp7a1-R	GTCCGGATATTCAAGGATGCA
Cyp27a1-F	GCCTTGCACAAGGAAGTGACT
Cyp27a1-R	CGCAGGGTCTCCTTAATCACA
Cyp8b1-F	GGCTGGCTTCCTGAGCTTATT
Cyp8b1-R	ACTTCCTGAACAGCTCATCGG
Cyp7b1-F	GAAAACTCTTCAAAGGCAACATGG
Cyp7b1-R	ACTGGAAAGGGTTCAGAACAAATG
Cyp2c70-F	TGGCTTTCTCAGCAGGAAGAA
Cyp2c70-R	AACTGGCTTGGTGTCGATGT
Cpt2-F	CCTGCTCGCTCAGGATAAACA
Cpt2-R	GTGTCTTCAGAAACCGCACTG
Ehhadh-F	CCAATGCAAAGGCTCGTGTT
Ehhadh-R	GGTAGAAGCTGCGTTCCTCTTG
Hadha-F	TGCATTTGCCGCAGCTTTAC
Hadha-R	GTTGGCCCAGATTTCGTTCA
Pck1-F	GTCTGGCTAAGGAGGAAGGG
Pck1-R	CAATGTCATCGCCCACACAT
G6pc-F	GGAGTCTTGTCAGGCATTGCTG
G6pc-R	AAGTCCACAGGAGGTCCACCC
Pcx-F	TTCTGGGGCCAATGACCTC
Pcx-R	TTATACTCCAGACGCCGGAC
Fbp-F	CCAATGTGACTGGGGATCAAG
Fbp-R	TGGTTCCGATGGACACAAGG
Gck-F	TTTGCAACACTCAGCCAGAC
Gck-R	TTACACTGGCCTCCTGATGG
Plin2-F	CAGCCAACGTCCGAGATTG
Plin2-R	CACATCCTTCGCCCCAGTT
Bacteria	
A. muciniphila-F	AGTATCGAAAGATTA AAGCAGCAATGC
A. muciniphila-R	TCTTGTGGTACTATCTTTTTAATTTGCT

*The qRT-PCR results were normalized to *Tbp* mRNA expression for ileum samples, to *Hprt* mRNA expression for WAT and BAT tissues, and to *Ywhaz* mRNA expression for liver tissues.

Original uncropped blots presented in this study:

Fig. 1b: Actin

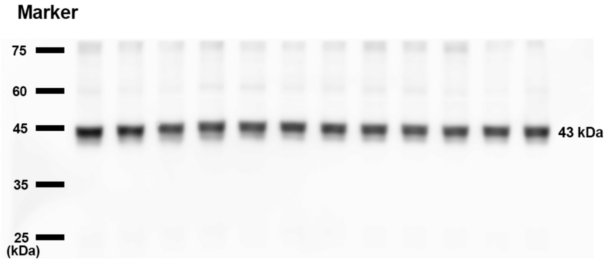

Fig. 1b: DUSP6

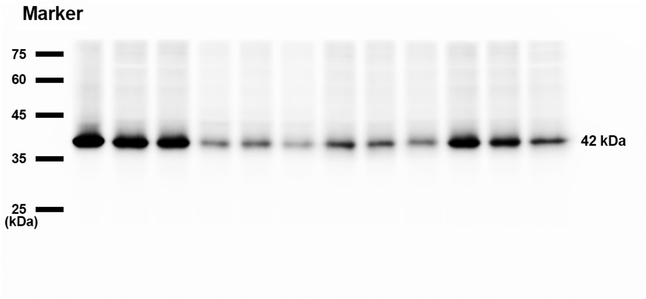

Supplement: Supplementary file 1 — Supplementary Information [file 41522_2024_495_MOESM1_ESM.pdf]
