# Supplementary material for: Intestinal dual-specificity phosphatase 6 regulates the cold-induced gut microbiota remodeling to promote white adipose browning
Source: NPJ Biofilms Microbiomes. 2024 Mar 13;10:22. doi: 10.1038/s41522-024-00495-8 (PMC10937957; doi:10.1038/s41522-024-00495-8)
Supplement: Supplementary file 2 — Reporting summary [file 41522_2024_495_MOESM2_ESM.pdf]

nature portfolio

Corresponding author(s): Jhen-Wei Ruan

Last updated by author(s): Feb 25, 2024

Reporting Summary

Nature Portfolio wishes to improve the reproducibility of the work that we publish. This form provides structure for consistency and transparency in reporting. For further information on Nature Portfolio policies, see our Editorial Policies and the Editorial Policy Checklist.

Statistics

For all statistical analyses, confirm that the following items are present in the figure legend, table legend, main text, or Methods section.

n/a	Confirmed	
☐	☒	The exact sample size (*n*) for each experimental group/condition, given as a discrete number and unit of measurement
☐	☒	A statement on whether measurements were taken from distinct samples or whether the same sample was measured repeatedly
☐	☒	The statistical test(s) used AND whether they are one- or two-sided<br>*Only common tests should be described solely by name; describe more complex techniques in the Methods section.*
☒	☐	A description of all covariates tested
☐	☒	A description of any assumptions or corrections, such as tests of normality and adjustment for multiple comparisons
☐	☒	A full description of the statistical parameters including central tendency (e.g. means) or other basic estimates (e.g. regression coefficient) AND variation (e.g. standard deviation) or associated estimates of uncertainty (e.g. confidence intervals)
☐	☒	For null hypothesis testing, the test statistic (e.g. *F*, *t*, *r*) with confidence intervals, effect sizes, degrees of freedom and *P* value noted<br>*Give P values as exact values whenever suitable.*
☒	☐	For Bayesian analysis, information on the choice of priors and Markov chain Monte Carlo settings
☒	☐	For hierarchical and complex designs, identification of the appropriate level for tests and full reporting of outcomes
☐	☒	Estimates of effect sizes (e.g. Cohen's *d*, Pearson's *r*), indicating how they were calculated

Our web collection on statistics for biologists contains articles on many of the points above.

Software and code

Policy information about availability of computer code

Data collection

For qPCR analysis, Ct value were collected using MyGo PCR systems from IT-IS Life Science Ltd.
For immunoblot analysis, the luminescence intensity was detected by the ImageQuant LAS 4000 mini imaging system (GE Healthcare Life Sciences).
For histology, images were visualized by Olympus BX43 microscope.
For 16s sequencing, 16s rRNA amplicons were sequenced on an Illumina Miseq platform.
For metabolomics, cecal metabolome was profiled by using Capillary Electrophoresis Time-of-Flight Mass Spectrometry (CE-TOFMS). Chromatography was carried out with fused silica capillary (50 µm × 80 cm)
For LC-MS/MS, samples were analyzed through Agilent 1200 Series Gradient HPLC System and API 5000™ LC/MS/MS System for collection. Chromatography was carried out with a Phenomenex bioZen™ 2.6 µm Glycan LC Column (100 mm × 2.1mm).

Data analysis

GraphPad Prism versions 9.3.0 was used to perform statistical analysis, as indicated in methods.
For qPCR analysis, relative gene expression were analysis through 2^-delta delta Ct method on Microsoft Excel 365.
For immunoblot analysis, quantification of proteins on immunoblots was performed with ImageJ software.
For histology, images were analyzed using Capture2.2 software.
For 16s microbiota analysis, raw sequences was performed using QIIME2 v2020.8. Amplicon sequence variant (ASVs) were analyzed in R studio to calculate alpha/beta diversity and network analysis. The network was visualized by Cytoscape software.
For RNA-seq analysis, REACTOME enrichment analysis was performed by the Database for Annotation, Visualization and Integrated Discovery (DAVID).
For LC-MS/MS, multiple reaction monitoring (MRM) analyses were performed on a API 5000™ LC/MS/MS System with Analyst® Software.

For manuscripts utilizing custom algorithms or software that are central to the research but not yet described in published literature, software must be made available to editors and reviewers. We strongly encourage code deposition in a community repository (e.g. GitHub). See the Nature Portfolio guidelines for submitting code & software for further information.

Data

Policy information about availability of data

All manuscripts must include a data availability statement. This statement should provide the following information, where applicable:

- Accession codes, unique identifiers, or web links for publicly available datasets
- A description of any restrictions on data availability
- For clinical datasets or third party data, please ensure that the statement adheres to our policy

The raw data of 16S rDNA sequencing has been deposited to NCBI Sequence Read Archive (SRA) under the following BioProject ID: PRJNA798518. This study does not report any original code. Any additional information required to reanalyze the data reported in this paper is available from the lead contact upon request. There is no custom code used in this manuscript.

Research involving human participants, their data, or biological material

Policy information about studies with human participants or human data. See also policy information about sex, gender (identity/presentation), and sexual orientation and race, ethnicity and racism.

Reporting on sex and gender	Research does not include human participants, their data, or biological material.
Reporting on race, ethnicity, or other socially relevant groupings	Research does not include human participants, their data, or biological material.
Population characteristics	Research does not include human participants, their data, or biological material.
Recruitment	Research does not include human participants, their data, or biological material.
Ethics oversight	Research does not include human participants, their data, or biological material.

Note that full information on the approval of the study protocol must also be provided in the manuscript.

Field-specific reporting

Please select the one below that is the best fit for your research. If you are not sure, read the appropriate sections before making your selection.

☒ Life sciences ☐ Behavioural & social sciences ☐ Ecological, evolutionary & environmental sciences

For a reference copy of the document with all sections, see nature.com/documents/nr-reporting-summary-flat.pdf

Life sciences study design

All studies must disclose on these points even when the disclosure is negative.

Sample size	No statistical method was used to predetermine sample size, but our sample size were similar to those reported in our previous publication (Ruan et al., PMID: 27892926)
Data exclusions	1. Mouse experiments: Mice displaying unusual behavior or unexpected weakness were excluded from further investigation.<br>2. qRT-PCR analysis: RNA samples with inadequate quantity or purity were not included in cDNA preparation.<br>3. LC-MS/MS analysis: Serum samples showing significant hemolysis were excluded from further investigation.
Replication	Every experiment in this study was extensively validated using multiple animals (ranging from 3 to 10 per group). The figure legends provided comprehensive information regarding replication and sample sizes. Histology analyses were conducted on every sample in the specified experiment, and the data was represented by an image from a single sample.
Randomization	CONV-R and germ-free mice were procured from the National Laboratory Animal Center, Taiwan. The mice were grouped through randomization, ensuring an equal distribution of body weight, and were accommodated in cages, with each cage housing a minimum of three but no more than five mice.
Blinding	The investigators were not blinded to allocation during experiments and data analysis. All measurements were conducted without bias from the investigators between the groups.

Reporting for specific materials, systems and methods

We require information from authors about some types of materials, experimental systems and methods used in many studies. Here, indicate whether each material, system or method listed is relevant to your study. If you are not sure if a list item applies to your research, read the appropriate section before selecting a response.

Materials & experimental systems

n/a	Involved in the study
☐	☒ Antibodies
☐	☒ Eukaryotic cell lines
☒	☐ Palaeontology and archaeology
☐	☒ Animals and other organisms
☒	☐ Clinical data
☒	☐ Dual use research of concern
☒	☐ Plants

Methods

n/a	Involved in the study
☒	☐ ChIP-seq
☒	☐ Flow cytometry
☒	☐ MRI-based neuroimaging

Antibodies

Antibodies used

Antibody information is supplied with antibody name, supplier name and catalog number/lot number.
1. Anti-DUSP6 antibody, Abcam, ab76310/GR222129-16
2. Anti-Actin Antibody, clone C4, Sigma-Aldrich, MAB1501/3282532
3. Anti-UCP1 antibody, Abcam, ab10983/GR3402177-1
4. HRP Donkey anti-rabbit IgG Antibody, Biolegend, 406401/B284927
5. HRP Donkey anti-mouse IgG Antibody, Biolegend, 405306/B287024

Validation

All antibodies were commercially available. Validation and citation were referred to the manufacture's websites, as followed:

1. Anti-DUSP6 antibody
-Validation: 'Knockout validated' on western blots.
-Citation: Schmidt C et al., Front Immunol, 9, 2386, 2018 Reproduced under the Creative Commons license http://creativecommons.org/licenses/by/4.0/
-Website: https://www.abcam.com/dusp6-antibody-epr129y-ab76310.html

2. Anti-Actin Antibody, clone C4
-Validation: It is routinely evaluated by western Blot on A431 lysates.
-Citation: Varela, A; Piperi, C; Sigala, F; Agrogiannis, G; Davos, CH; Andri, MA; Manopoulos, C; Tsangaris, S; Basdra, EK; Papavassiliou, AG Scientific reports 5 13461 2015
-Website: https://www.merckmillipore.com/TW/zh/product/Anti-Actin-Antibody-clone-C4,MM_NF-MAB1501

3. Anti-UCP1 antibody
-Validation: It is validated by western blot on rat brown fat lysate and by immunohistochemistry staining on murine inguinal white adipose tissue.
-Citation: Cao X, Shi TT, Zhang CH, Jin WZ, Song LN, Zhang YC, Liu JY, Yang FY, Rotimi CN, Xu A, Yang JK. ACE2 pathway regulates thermogenesis and energy metabolism. Elife. 2022 Jan 11;11:e72266. doi: 10.7554/eLife.72266. Erratum in: Elife. 2022 Mar 22;11: PMID: 35014608; PMCID: PMC8776250.
-Website: https://www.abcam.com/products/primary-antibodies/ucp1-antibody-ab10983.html

4. HRP Donkey anti-rabbit IgG Antibody
-Validation: This product lot has passed BioLegend's QC testing and is certified for use. For details on QC testing view our page at biolegend.com/en-gb/quality-control.
-Citation: Ram AK, Vairappan B, Srinivas BH. Nimbolide inhibits tumor growth by restoring hepatic tight junction protein expression and reduced inflammation in an experimental hepatocarcinogenesis. World J Gastroenterol. 2020 Dec 7;26(45):7131-7152. doi: 10.3748/wjg.v26.i45.7131. PMID: 33362373; PMCID: PMC7723674.
-Website: https://www.biolegend.com/en-gb/products/hrp-donkey-anti-rabbit-igg-minimal-x-reactivity-2264?GroupID=BLG3472

5. HRP Donkey anti-mouse IgG Antibody
-Validation: This product lot has passed BioLegend's QC testing and is certified for use. For details on QC testing view our page at biolegend.com/ja-jp/quality-control.
-Citation: Winters T, McNicoll F, Jessberger R. Meiotic cohesin STAG3 is required for chromosome axis formation and sister chromatid cohesion. EMBO J. 2014 Jun 2;33(11):1256-70. doi: 10.1002/embj.201387330. Epub 2014 May 5. PMID: 24797474; PMCID: PMC4198028.
-Website: https://www.biolegend.com/ja-jp/products/hrp-goat-anti-mouse-igg-minimal-x-reactivity-1395?GroupID=BLG2049

Eukaryotic cell lines

Policy information about cell lines and Sex and Gender in Research

Cell line source(s)	Mouse 3T3-L1 adipocytes
Authentication	The 3T3-L1 mouse cell line was obtained from ZenBio Inc. and was authenticated.
Mycoplasma contamination	The 3T3-L1 mouse cell line was verified to be free of mycoplasma contamination by ZenBio Inc.
Commonly misidentified lines (See ICLAC register)	Non.

Animals and other research organisms

Policy information about studies involving animals; ARRIVE guidelines recommended for reporting animal research, and Sex and Gender in Research

Laboratory animals	5-to-6-week-old C57BL/6J mice were purchased from National Laboratory Animal Center (NLAC), NARLabs, Taiwan. Mice were housed with a 13-hour light/11-hour dark cycle (light from 7 A.M. to 8 P.M.) at 22℃ and free access to autoclaved food and water. They were kept in individually ventilated cages (IVC) under specific pathogen-free conditions. Germ-free mice were kept in the IsoCage Bioexclusion System (Tecniplast) under sterile conditions.
Wild animals	No wild animals were used in this study.
Reporting on sex	Male mice were used in this study.
Field-collected samples	No field-collected samples were used in this study.
Ethics oversight	All animal experiments were approved by the Institutional Animal Care and Use Committee (IACUC) at NCKU.

Note that full information on the approval of the study protocol must also be provided in the manuscript.

Plants

Seed stocks	*Report on the source of all seed stocks or other plant material used. If applicable, state the seed stock centre and catalogue number. If plant specimens were collected from the field, describe the collection location, date and sampling procedures.*
Novel plant genotypes	*Describe the methods by which all novel plant genotypes were produced. This includes those generated by transgenic approaches, gene editing, chemical/radiation-based mutagenesis and hybridization. For transgenic lines, describe the transformation method, the number of independent lines analyzed and the generation upon which experiments were performed. For gene-edited lines, describe the editor used, the endogenous sequence targeted for editing, the targeting guide RNA sequence (if applicable) and how the editor was applied.*
Authentication	*Describe any authentication procedures for each seed stock used or novel genotype generated. Describe any experiments used to assess the effect of a mutation and, where applicable, how potential secondary effects (e.g. second site T-DNA insertions, mosiacism, off-target gene editing) were examined.*
